# ApoE4 requires lipidation enhancement to resolve cellular lipid and protein abnormalities following NPC1 inhibition

**DOI:** 10.1038/s41598-025-96531-4

**Published:** 2025-04-29

**Authors:** Erika Di Biase, Kyle J. Connolly, Ingrid Crumpton, Oliver Cooper, Penelope J. Hallett, Ole Isacson

**Affiliations:** Neuroregeneration Institute, McLean Hospital, Harvard Medical School, Belmont, MA 02478 USA

**Keywords:** ApoE, Alzheimer’s risk variant, NPC1, Lipid transport, Human cellular platform, Amyloid, Triglycerides, Cholesterol, ApoE4 drug therapy, Astrocyte, Fibroblast., Cell biology, Genetics, Neuroscience

## Abstract

Apolipoprotein E (ApoE) variants are central to Alzheimer’s disease (AD), Lewy body dementia (LBD) and Niemann-Pick disease type C (NPC). The ApoE4 variant elevates AD risk by 3-15-fold. ApoE’s normal function in lipid transport is known. The question remains how different ApoE isoforms cause cellular pathogenesis. We determined the effects of ApoE isoforms on lipid accumulation induced by inhibiting the endo-lysosomal cholesterol transporter NPC1. In human fibroblasts and astrocytes, NPC1 inhibition caused a 4-fold cholesterol accumulation and mis-localization with altered cholesterol sensing and increased synthesis of cholesterol and triglycerides. Total APP, APP C-terminal fragments (CTF) and BACE1 levels increased 3-fold. Remarkably, the intracellular neutral lipids co-localized with APP and APP C-terminal fragments. ApoE2 and ApoE3, but not ApoE4, reduced intracellular cholesterol levels by 67% and 62%, respectively, normalized APP, BACE, CTF, and improved cell survival. ApoE4 combined with a synthetic lipopeptide, which increased the proportion of large lipidated ApoE4 particles, corrected these abnormalities. This highlights ApoE in lipid pathogenesis and targeting ApoE4 lipidation to restore ApoE4 function.

**Supplementary Information:**

The online version contains supplementary material available at 10.1038/s41598-025-96531-4.

## Introduction

Lipid trafficking and metabolism play significant roles in the etiopathogenesis of age-related chronic diseases, such as vascular and cognitive decline^1,2^. How lipid dysregulation impacts human cell biology and function in the nervous system is relevant to our understanding of neurodegeneration in age-related diseases such as late-onset Alzheimer’s (LOAD), Parkinson’s, and Lewy body dementia (LBD).

The apolipoprotein E (ApoE) 4 variant is quantitively the major genetic risk factor for the development of LOAD, LBD and related dementias^3–7^. Individuals heterozygous for ApoE4 have about 3-fold higher risk, while homozygous have around 15-fold higher risk to develop dementia than those carrying a ApoE3 allele^4^. Systemically, ApoE’s function is well described in the vascular and nervous systems^8^. Mechanistically, ApoE transports lipids such as cholesterol, triglycerides, and phospholipids across various cells and tissues^9,10^. In non-brain tissues, ApoE is predominantly synthesized and secreted by the liver. In the brain, the supply of ApoE is primarily derived from astroglia. Under circumstances of injury or cellular lipid stress, microglia and neurons can respond by increased ApoE expression^11,12^. The human ApoE2, ApoE3, and ApoE4 isoforms differ by single amino acid variations at positions 112 and 158 ^9^. ApoE3 is the most common isoform and has a cysteine residue at position 112 and an arginine residue at 158. ApoE2, the rarest isoform, has cysteine at both positions 112 and 158, whereas ApoE4 has arginine residues at both positions. Functionally, ApoE4 has been shown to be relatively less lipidated than ApoE2 and ApoE3^13,14^ which may relate to deleterious effects^15–17^. The lower level of ApoE4 lipidation suggests that increasing ApoE lipidation may be a viable therapeutic avenue for AD and other neurological disorders^6,18^.

Mutations in genes connected with ApoE biological function such as ABCA1, which loads cholesterol onto ApoE, or other cholesterol transporters within cells (such as NPC1), also increase the risk of neurological pathologies^19–22^. Genome-wide association studies (GWAS) have identified multiple lipid metabolism-related risk alleles for AD^23^. Overall, these genetic data support a central lipid dyshomeostasis hypothesis for the causes of age-related dementia development in AD, LBD, and related dementias^1,24–30^. Mutations in the NPC1 gene are responsible for the endo-lysosomal cholesterol transport deficits leading to Niemann-Pick type C1 disease (NPC). Interestingly, patients with NPC also have brain accumulation of β-amyloid plaques^31^ similar to AD^22,32^. NPC patients exhibit earlier symptom onset and more profound cognitive deterioration, which correlates with the ApoE ε4 allele count^33^, further reinforcing the interactions between ApoE4, NPC1 dysfunction and dementia^33–37^. Despite the significant strengths and evidence of the lipid dyshomeostasis hypothesis involving these genes and their classic biology, the collective impact of these genes on lipid transport, protein processing, cellular integrity, brain inflammation and disease progression is not understood. These findings are likely not limited to lysosomal lipid disturbance for NPC1, but generally can relate to lysosomal and lipid abnormalities in several other neurodegenerative diseases. LBD, for example has the strongest genetic risk factors in variants of ApoE and glucocerebrosidase (GBA1), which is the enzyme that when lost increases glycosphingolipid levels. Animal model studies provide evidence that the broader lysosomal deficiencies^38,39^ can phenocopy Parkinson’s disease (PD), LBD and AD clinical biomarkers. The current study can therefore be seen in a series of in-depth exploration for lipid abnormalities that drive many neurodegenerative diseases with significant healthcare impacts^1,24,30^. Cholesterol load has been seen in NPC1 patients and in many cases of AD/dementia syndromes^40,41^. While this study is focused on the specific in vitro platform, the results reveal important cell biological connections between cholesterol transport, lysosomal deficits and cellular responses that underlie many of these diseases.

In the data reported here, a human cellular in vitro platform system disrupting endo-lysosomal cholesterol metabolism was generated to identify the downstream cellular and molecular consequences of intracellular cholesterol and lipid accumulation. The experiments show the functional effects of recombinant human ApoE isoforms (2, 3 and 4) in cells with pharmacologically induced intracellular cholesterol and lipid load. Reduced ApoE4 cholesterol efflux and associated cellular functions relative to equimolar doses of ApoE2 and ApoE3 was associated with cell phenotypic changes and cell death in human fibroblasts and astrocytes. In a proof of concept study in vitro using an amphipathic peptide combined with ApoE4 supplementation, ApoE4 function improved cholesterol efflux and rescued all phenotypic changes observed following NPC1 inhibition in these cellular model systems.

## Results

### Alteration of cholesterol transport and synthesis in the NPC1-inhibited human fibroblasts platform

Fibroblasts are readily available cells, easily handled for high-throughput experiments. In this cellular biological platform, four human fibroblast lines derived from healthy subjects (HS) were used, here referred to as HS21, HS23, HS22, HS28 and line #1, #2, #3, #4, respectively. Firstly, the ApoE genotype and endogenous expression were evaluated in these cells. Two SNPs, rs429358 and rs7412, define ApoE alleles^42^ (Supplementary Fig. 1A) and gene sequencing revealed HS21 (line #1) as ApoE3/3, HS23 (line #2) as ApoE3/4 and HS22 (line #3), HS28 (line #4) as ApoE2/3 (Supplementary Fig. 1B). Regardless of ApoE genotype, fibroblasts’ ApoE mRNA levels were barely detectable and 200-fold lower than the hiPSC derived astrocyte line used as positive control of ApoE expression (Supplementary Fig. 1D). In addition, the ApoE protein levels were undetectable as measured by Western Blot (WB) in fibroblasts lysates (Supplementary Fig. 1C), showing that the endogenous ApoE levels of human fibroblasts where lower than the assay limit of detection.

**Fig. 1:**
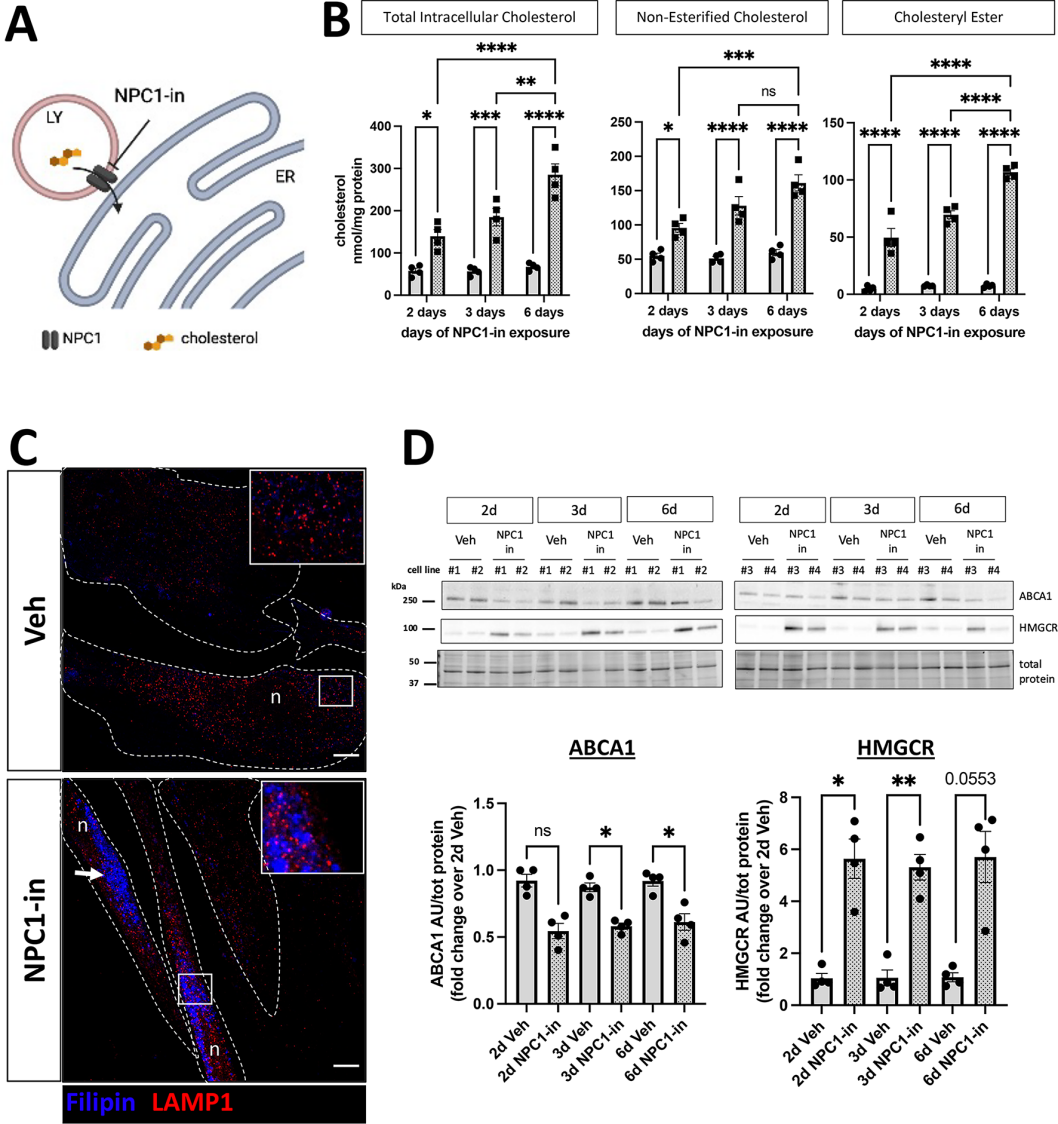
NPC1 inhibition induces intracellular cholesterol accumulation in human fibroblasts. (**A**) Scheme of the inhibition of the late endo-lysosomal (LY) cholesterol transporter NPC1 mediated by U18666A. In this way cholesterol cannot be transported to the endoplasmic reticulum (ER) and accumulates into the lysosomes. (**B**) Total, esterified and free cholesterol levels measured in cell lysates by Amplex Red cholesterol assay after 2, 3 and 6 days of NPC1-inhibition. Each dot represents one cell line, and the values are the mean ± SEM of three independent experiments. (**C**) Representative confocal images of filipin (blue) and LAMP1 (red) immunosignals. 100X magnification, scale bar: 10 µm. Top-right Inserts are 3-fold magnification of the respective region of interest (ROI). 2 coverslips per condition with 10 images acquired from each coverslip, from 2 independent experiments were analyzed. (**D**) Representative WB images (top) and relative quantification (bottom) of proteins involved in cholesterol synthesis: 3-hydroxy-3-methylglutaryl-CoA reductase (HMGCR) and ATP-binding cassette transporter A1 (ABCA1) mediating lipid efflux. Proteins detected by WB were normalized to total protein levels at the 50-37kDa molecular weight range. Each dot represents one cell line (four individual cell lines total), and the values are the mean ± SEM of three independent experiments. Pairwise one way ANOVA with post-hoc Tukey for multiple comparison, *p  < 0.05, **p  < 0.01, ****p  < 0.0001. Veh: vehicle (PBS). NPC1-in: NPC1 inhibitor (U18666A).

In order to induce a primary intracellular cholesterol impairment, fibroblasts were exposed to the U18666A drug, a potent inhibitor of the lysosomal transporter NPC1^43^, mimicking the Niemann-Pick disease type C1. The U18666A mechanism of action is described in Fig. 1A: cholesterol derived from receptor-mediated uptake of lipoproteins and from plasma membrane turnover is exported by NPC1, and U18666A inhibits this process at the endo-lysosomal level. Accordingly, the exposure to the NPC1 inhibitor induced a progressive accumulation of total intracellular cholesterol, either free, not-esterified cholesterol and cholesteryl-ester (Fig. 1B). In NPC1 inhibited cells [hereafter referred to as NPC1(-) in the text, and NPC1-in in the Figures] the filipin (free cholesterol probe) signal significantly increased, and partially co-localized with LAMP1 immunosignal showing the accumulation of free cholesterol into late endo-lysosomes (Fig. 1C and Supplementary Fig. 2). To better understand the cell response to the cholesterol accumulation, the expression of proteins specifically involved in cholesterol metabolism was evaluated. This included the ATP-binding cassette transporter A1 (ABCA1) mediating cholesterol efflux to the outside the cell through ApoE lipoproteins. One of the key enzymes involved in the *de novo* cholesterol synthesis, 3-hydroxy-3-methylglutaryl coenzyme A reductase (HMGCR)^44^ was also measured. Interestingly, after 2, 3 and 6 days of NPC1 inhibition the cells showed a significant reduction of ABCA1 levels, and increased levels of HMGCR enzyme (Fig. 1D). This suggested that the cells upregulated the pathway to actively synthesize more cholesterol and downregulated the factors required for cholesterol expulsion, perhaps because of intracellular mis-localization of cholesterol after the NPC1 inhibition, causing loss of appropriate sensing by the sterol regulatory element-binding protein (SREBP) molecular machinery^45–47^.

### Neutral lipids increase in NPC1 inhibited cells due to de novo synthesis

**Fig. 2:**
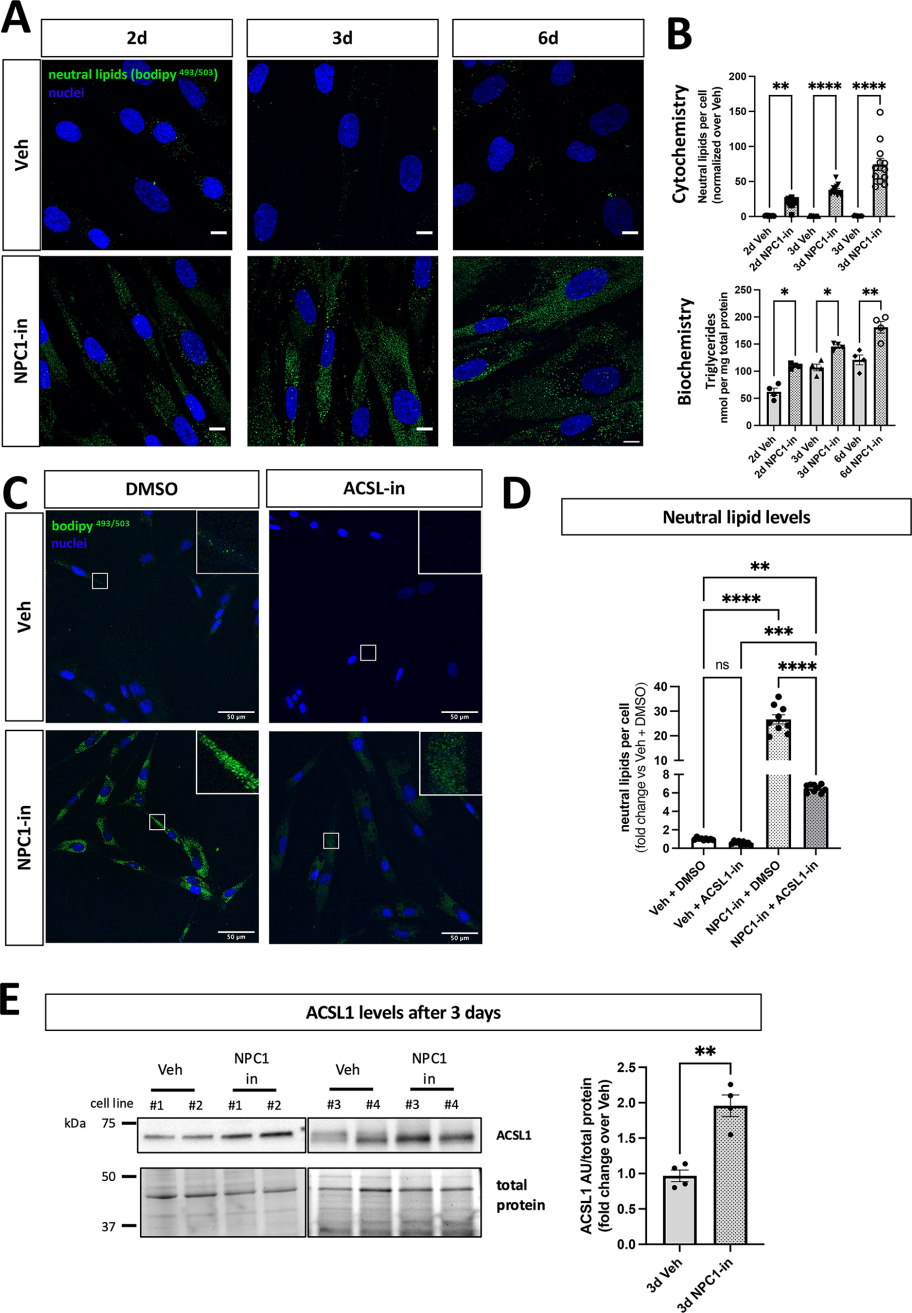
NPC1 inhibited fibroblasts accumulate lipid droplets and triglycerides. (**A**) Representative confocal images of bodipy^493/503^ positive neutral lipids (green) in cells challenged with NPC1 inhibitor for 2,3 and 6 days. Nuclei are in blue. 100X magnification, scale bar: 10 μm. The graph on the right shows the quantification of the area occupied by bodipy signal relative to the number of cell nuclei. 2 coverslips per condition with 10 images acquired from each coverslip, from 3 independent experiments were analyzed. (**B**) Triglycerides quantification in cell lysates. Each dot represents one cell line. The values are the mean ± SEM of 3 independent experiments. (**C**)^493/503^ staining in 3-day NPC1-inhibited fibroblasts treated with Triacsin C, the inhibitor of long fatty acyl CoA synthetase, ACSL1 (ACSL1-in) or vehicle (DMSO). 40X magnification, scale bar: 50 µm. Top-right Inserts are 4-fold magnification of the respective region of interest. (**D**) Quantification of the area of bodipy signal relative to the number of cell nuclei. 2 coverslips per condition with 10 images acquired from each coverslip, from 2 independent experiments were analyzed. (**E**) Representative WB images and relative quantification of ACSL1. Proteins detected by WB were normalized to total protein levels at the 50-37kDa molecular weight range. Each dot represents one cell line, and the values are the mean ± SEM of 3 independent experiments. Pairwise one way ANOVA with post-hoc Tukey for multiple comparison (**B**,**D**), Pairwise Student t-test (**E**), *p < 0.05, **p   < 0.01, ****p   < 0.0001. Veh: vehicle (PBS). NPC1-in: NPC1 inhibitor (U18666A).

The intracellular neutral lipids levels were further evaluated by quantifying presence of lipids droplets using bodipy^493/503^^48^. Fibroblasts showed basal level of lipid droplets, which progressively accumulated upon NPC1 inhibition (Fig. 2A, B).

Cholesteryl-ester and triglycerides are the main neutral lipids stored in lipid droplets. In agreement with an overall accumulation of lipid droplets, a significant cholesteryl-ester increase was observed in NPC1(-) cells (Fig. 1B). NPC1(-) cells also showed elevations of triglyceride levels (Fig. 2B). To understand the molecular mechanism underlying lipid droplets accumulation upon NPC1 inhibition, NPC1(-) cells were treated with Triacsin C, the inhibitor of the long fatty acyl CoA synthetase (ACSL)^49^, responsible for the *de novo* synthesis of new lipid droplets. ACSL1-inhibition significantly lowered the amount of lipid droplets in NPC1(-) cells (Fig. 2C, D), which however did not reach control levels. Interestingly, following NPC1 inhibition the cells expressed 1.75-fold higher ACSL1 protein than control cells (Fig. 2E), supporting an active neutral lipid biogenesis. It has been previously reported that NPC1 inhibition increases the demand of the autophagic system, which could contribute to the accumulation of undegraded intracellular components^50^. Consistent with this, a significant reduction of the autophagic flux was observed in NPC1(-) cells (Supplementary Fig. 3). Two major autophagic markers, p62 and LC3 II, were evaluated in the presence of rapamycin or bafilomycin, an activator and inhibitor of the autophagic flux, respectively. NPC1-inhibition induced an increase of both markers suggesting an accumulation of autophagosomes (high LC3 II) and insufficient degradative capacity (high p62), which was similar to bafilomycin exposure.

### ApoE rescues the viability and lipid phenotype of NPC1(-) cells with ε2= ε3> ε4

**Fig. 3:**
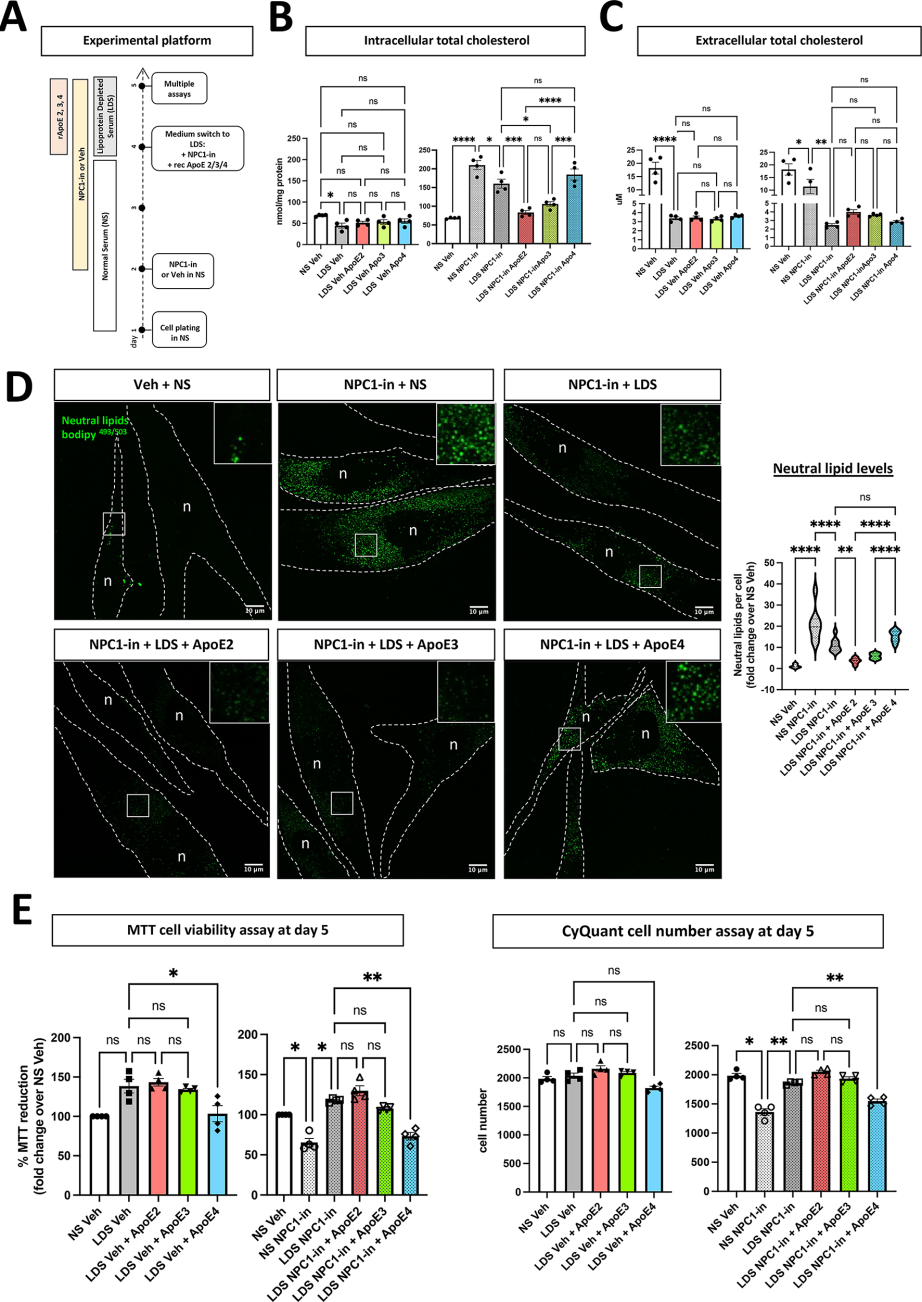
A cellular platform for investigating ApoE related functions in lipid stress. Demonstration of the relative capacity of ApoE isoforms to reduce cholesterol and lipid droplets load in NPC1-inhibited fibroblasts (n = 4). The results showed the biological capacity in the following order: ApoE2 = ApoE3 > ApoE4. (**A**) Scheme of the experimental workflow: NPC1-inhibited fibroblasts were cultured for 2 days in medium containing 10% FBS (normal serum, NS) which was substituted with lipoprotein depleted serum (LDS) plus equimolar concentration of recombinant apolipoproteins E (ApoE 2, 3, 4) and cultured one additional day before cell assays. (**B**,**C**) Intracellular (**B**) and extracellular (**C**) cholesterol measurement in all the experimental conditions. Each dot represents one cell line, and the values are the mean ± SEM of 3 independent experiments. (**D**) Representative confocal images of bodipy^493/503^ positive neutral lipids (green) and relative signal quantification per cell number. ×100 magnification, scale bar 10 µm. Top-right inserts are 3-fold magnification of the respective ROI. 2 coverslips per condition with 10 images acquired from each coverslip, from 2 independent experiments were analyzed. n: nucleus. The cell boundaries are highlighted by white dashed lines. (**E**) Viability assays. Each dot represents one cell line, and the values are the mean ± SEM of 3 independent experiments. Pairwise one-way ANOVA with post-hoc Tukey for multiple comparison *p < 0.05, **p < 0.01, ****p < 0.0001. Veh: vehicle (PBS). NPC1-in: NPC1 inhibitor (U18666A).

Our key experimental biological hypothesis relates to the interaction and responses of apolipoproteins during lipid challenges that simulate age and neurodegenerative disease. Given that the results show that inhibition of the NPC1 transporter caused an accumulation of cholesterol and neutral lipids in fibroblasts, we introduced an experimental paradigm to test the effect of ApoE variants in this cellular system. These experiments established the distinctive functional roles of different ApoE isoforms in NPC1 inhibited fibroblasts. Recombinant human ApoEs were aimed at investigating isoform-specific variations in lipid lowering capacity in cholesterol accumulating cells. First, fibroblasts were incubated for two days with the NPC1 inhibitor in complete growth medium supplemented with 10% fetal bovine serum (FBS), referred to as normal serum (NS) (Fig. 3A). To avoid the influence of lipoproteins from serum source, NS was replaced with lipoprotein-depleted serum (LDS). Concurrently, lipid-free recombinant apolipoproteins ApoE2, ApoE3, or ApoE4 were introduced into the culture at a concentration of 10 µg/mL^51–53^, in both the presence and absence of the NPC1 inhibitor, for an additional day. The resulting intracellular and extracellular cholesterol levels were quantified (Fig. 3B, C). Substitution of NS with LDS in NPC1(-) cells led to a decrease in the average extracellular cholesterol concentration by 84.4 ± 6.2%, attributable to the removal of extracellular cholesterol bound to serum lipoproteins (Fig. 3C). This resulted in a 23.3 ± 9.9% reduction in intracellular cholesterol levels in cells cultured with LDS and the NPC1 inhibitor (LDS/NPC1(-)) compared to those cultured with NS and the NPC1 inhibitor (NS/NPC1(-)) (Fig. 3B). The addition of different human ApoE isoforms to the media notably impacted intracellular cholesterol levels. Cells treated with ApoE2 exhibited the most pronounced decrease in cholesterol accumulation, showing a reduction of 47.8 ± 4.5% versus LDS/NPC1(-) cells. On the other hand, ApoE4 supplementation resulted in no detectable change in intracellular cholesterol when compared to LDS/NPC1(-) cells. ApoE3 produced an intermediate effect, reducing intracellular cholesterol by 33.3 ± 9.0%, which was not statistically different from the reduction observed with ApoE2 treatment. Corroborating these observations, filipin staining was more intense and widespread in NPC1(-) cells treated with ApoE4 relative to those treated with ApoE2 or ApoE3 (Supplementary Fig. 4). Additionally, there was a noticeable trend toward increased extracellular cholesterol levels in the presence of ApoE2 and ApoE3 (Fig. 3C). Further analysis was therefore conducted by calculating the ratios of intracellular to extracellular cholesterol concentrations (Table 1), enabling the comparison of the different experimental conditions. In LDS/NPC1(-) cells, the addition of ApoE2 and ApoE3 significantly lowered this ratio, whereas the ratio remained unaffected by the addition of ApoE4.

**Table 1:** Evaluation of differences in ApoE isoform performance on intracellular/extracellular cholesterol load. Using the cellular platform (see Fig. 3A), the capacity of the ApoE isoforms 2, 3 and 4 to restore the intracellular/extracellular lipid balance, is described as a ratio. The data for calculating the ratio between intracellular and extracellular cholesterol of the cell conditions are shown in Fig. 3B and C. Paired one-way ANOVA with post-hoc Tukey for multiple comparison (*n* = 3) ****p < 0.001.

Condition	Ratio
LDS, NPC1-in	66.06 ${\pm}$ 14.97
LDS, NPC1-in, ApoE 2	21.17 $\pm$ 3.95**** (vs. LDS, NPC1-in)
LDS, NPC1-in, ApoE 3	29.17 $\pm$ 4.12**** (vs. LDS, NPC1-in)
LDS, NPC1-in, ApoE 4	64.43 $\pm$ 8.02 (ns vs. LDS, NPC1-in)

Considering the neutral lipids accumulation occurring after NPC1 transporter inhibition, ApoE influence on neutral lipids levels was evaluated in addition to its effect on cholesterol load. The levels of bodipy^493/503^ positive lipid droplets in NPC1(-) cells exposed to ApoE2, 3 and 4 were measured. The replacement of NS with LDS significantly lowered the levels of neutral lipids in NPC1(-) cells (Fig. 3D). The addition of ApoE2 and ApoE3 further lowered the amount of lipid droplets close to that seen in NS/Veh cells. In contrast, ApoE4 receiving cells maintained neutral lipid levels significantly higher than ApoE2 and ApoE3, indistinguishable from the LDS/NPC1(-) condition, indicating a failure to reduce the lipid load accumulated in NPC1(-) cells.

Next, the impact of NPC1 inhibition and the effect of ApoE isoforms on cell viability was investigated. NS/NPC1(-) cells presented a significantly lower cellular viability than NS-Veh cells measured by MTT assay and by the cell number quantification (Fig. 3E). LDS/NPC1(-) cells receiving either ApoE2 and ApoE3 maintained their viability, and LDS/NPC1(-) cells receiving ApoE4 did not, showing a viability level similar to NS/NPC1(-), but actually worse viability than non-lipid depleted serum, suggesting that lipidation is critical for its non-toxic function. These data show that the addition of human recombinant ApoE4 failed to promote cell survival during lipid challenge produced by inhibition of NPC1 in the endo-lysosomal system.

### ApoE2 and ApoE3, but not ApoE4, reduced the NPC1-in induced elevation of full-length APP and C-terminal fragments

**Fig. 4:**
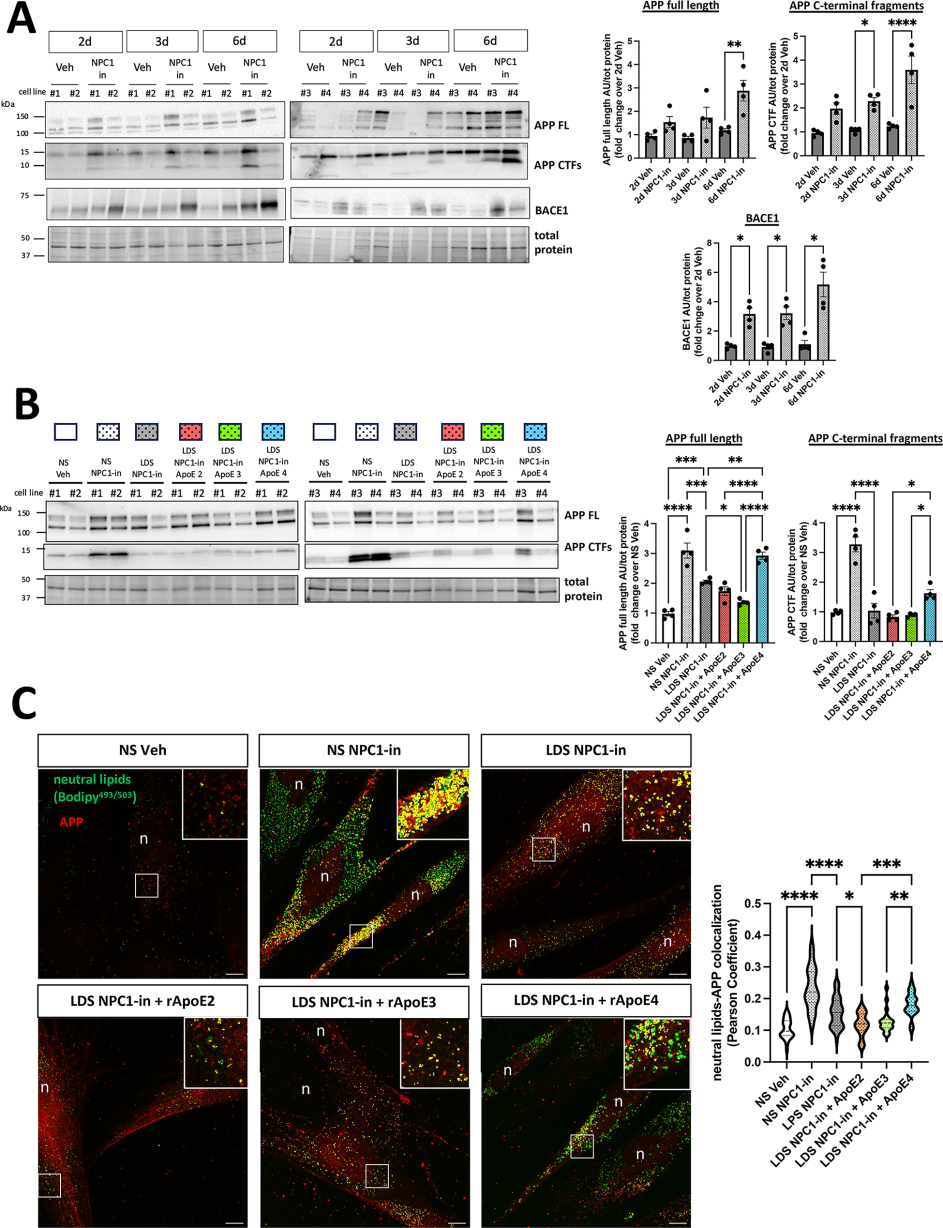
ApoE2 and 3, but not ApoE4, reduced the increased full-length APP and C-terminal fragments indued by NPC1 inhibition and altered cholesterol level. (**A**) Representative WB images and relative quantification of full-length (FL) APP, C-terminal fragments (CTFs) and Beta-Secretase 1 (BACE1) in fibroblasts after 2, 3 and 6 days (d) of NPC1 inhibition. Each dot represents one cell line, and the values are the mean ± SEM of 2 independent experiments. (**B**) Representative WB images and relative quantification of full-length (FL) APP and C-terminal fragments (CTFs) in NPC1-inhibited fibroblasts treated with ApoE isoforms following the scheme in Fig. 3A. Proteins detected by WB were normalized to total protein levels at the 50-37 kDa molecular weight range. Each dot represents one cell line, and the values are the mean ± SEM of 2 independent experiments. (**C**) Representative confocal images (maximal projections) of bodipy^493/503^ positive neutral lipids (green) and APP (red). APP and bodipy colocalizing pixels in yellow. Nuclei are in blue. 100X magnification, scale bar 10 μm. Top-right inserts are 3-fold magnification of the respective ROI. The graph on the right shows the Pearson coefficients of APP and bodipy colocalizing pixels in each image section. 3 coverslips per condition with 10 images acquired from each coverslip, from 2 independent experiments were analyzed. Pairwise one way ANOVA with post-hoc Tukey for multiple comparison (*p < 0.05, **p < 0.01, ****p < 0.0001). Veh: vehicle (PBS). NPC1-in: NPC1 inhibitor (U18666A). NS: normal serum (10% FBS). LDS: lipoprotein depleted serum.

The modification of intracellular cholesterol levels can alter the processing of the amyloid precursor protein (APP)^54–56^, however such findings are yet to be fully explored for neurodegenerative diseases. Increased levels of APP and C-terminal fragments have been associated with cellular stress and toxicity^57–59^. For that reason, we tested how cholesterol and neutral lipid dysregulation, resulting from NPC1 inhibition, can impact endogenous levels of full-length APP and its C-terminal fragments. In fibroblasts growing in medium with normal serum (NS), NPC1 inhibition induced an increase of both full-length APP and C-terminal fragments (Fig. 4A). Increased cholesterol levels have also been associated with elevated production of soluble Aβ42 and Aβ40 peptides^31,55,60–62^. To investigate these peptides, their presence was evaluated in the fibroblast platform, both in cell supernatants and lysates, using WB and Elisa methods. However, no signal was observed under any experimental condition, indicating that their levels were below the minimum level of detection for these assays (data not shown). Increased levels of Beta-Site APP Cleaving Enzyme 1 (BACE1) protein ($\:{\upbeta\:}$-secretase) have been associated with altered APP processing in Alzheimer’s diseases^63–65^. In the 2, 3, 6 days exposure of fibroblasts to NPC1 inhibitor there was a significant elevation of BACE1 levels (Fig. 4A), indicating that cholesterol and lipid accumulation directly influence APP processing.

Given the observed ApoE ability to reduce lipid load in NPC1(-) cells, the effects of ApoE isoforms on APP levels were also evaluated (Fig. 4B). Lipoprotein depletion (LDS) in NPC1(-) cells significantly reduced full-length APP and C-terminal fragments compared to NS/NPC1(-) cells. APP C-terminal fragments levels remained stable with the addition of recombinant ApoE2 and ApoE3, with ApoE3 further reducing the full-length form of the protein compared to LDS/NPC1(-) cells. Conversely, LDS/NPC1(-) cells receiving recombinant ApoE4 showed significantly higher levels of both full-length APP and C-terminal fragments compared to LDS/NPC1(-) cells. These findings show that the cholesterol and APP elevating effects of cellular endo-lysosomal inhibition of NPC1 is influenced and compensated by presence of ApoE. The relative competence of these biological effects was in the order of ApoE2 and ApoE3, compared to the less efficient ApoE4.

Next, the intracellular localization of APP and neutral lipids were evaluated based on the observation of the increase in APP and neutral lipids seen in NPC1 inhibited cells and the isoform dependent ApoE mediated reduction. Co-staining for APP and neutral lipids revealed an increased co-localization in NS/NPC1(-) cells compared to NS/Veh cells (Fig. 4C). The replacement of NS with LDS in NPC1(-) cells reduced the level of APP-neutral lipid co-localization. LDS/NPC1(-) cells receiving recombinant ApoE2 and ApoE3 further reduced APP and neutral lipid co-localization, while it remained significantly higher when ApoE4 was administered. Overall, these data demonstrate that ApoE2 and ApoE3 can improve and normalize APP processing in the presence of accumulated cellular lipids caused by NPC1 inhibition, whereas the ApoE4 isoform fails functionally in this cellular system.

### The apolipoprotein mimetic peptide 4F restored ApoE4 function in NPC1 inhibited fibroblasts

**Fig. 5:**
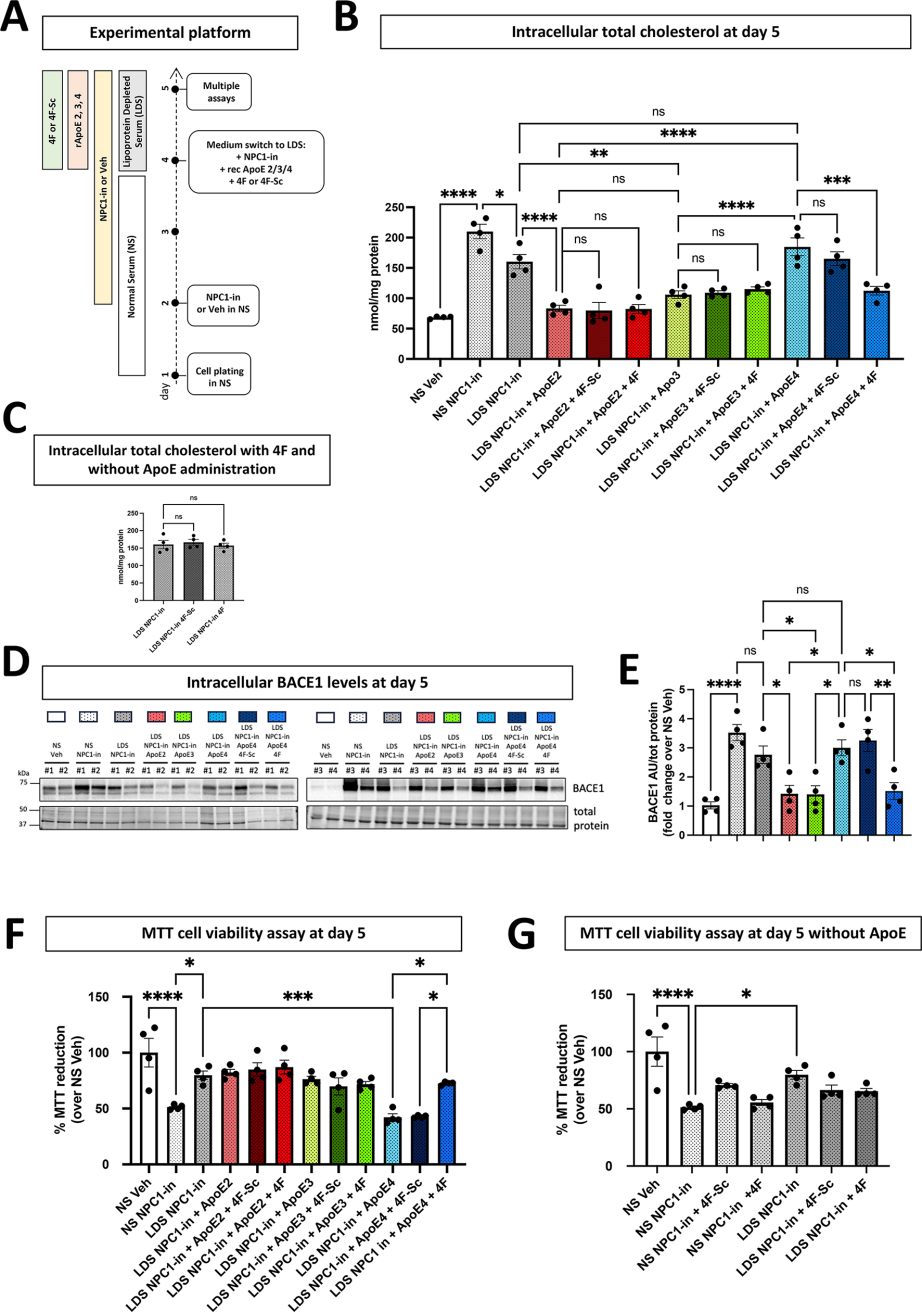
Description and data for the cellular experimental platform used with lipid stress evaluating the 4F lipopeptide to restore ApoE4 function in NPC1-inhibited fibroblasts. (**A**) Scheme of the experimental workflow: NPC1-inhibited fibroblasts cultured for 2 days in medium containing 10% FBS (normal serum, NS) were switched to the medium containing lipoprotein depleted serum (LDS) with the addition of equimolar concentrations of recombinant human apolipoproteins (ApoE 2, 3, 4) in the presence of 4F lipopeptide or scrambled 4F (4F-Sc) and cultured one additional day. (**B**,**C**) Intracellular cholesterol measurements. (**D**) Representative WB images of BACE1 protein. Each dot represents one cell line, and the values are the mean ± SEM of 3 independent experiments. (**E**) Relative quantification of BACE1 WB bands. BACE1 protein detected by WB was normalized to total protein levels at the 50-37 kDa molecular weight range. Each dot represents one cell line, and the values are the mean ± SEM of 2 independent experiments. (**F**,**G**) MTT viability assay. Each dot represents one cell line, and the values are the mean ± SEM of 3 independent experiments. Pairwise one way ANOVA with post-hoc Tukey for multiple comparison (*p < 0.05, **p < 0.01, ***p < 0.001, ****p < 0.0001). Veh: vehicle (PBS). NPC1-in: NPC1 inhibitor (U18666A).

In order to evaluate whether the human cellular platform can be employed for testing ApoE modifier molecules improving ApoE4 function, we measured the effectiveness of the short apolipoprotein mimetic peptide 4F in reversing the ApoE4 limitations in cholesterol transport and cell viability. 4F has been previously observed to increase ApoE lipidation and secretion^66^. We systematically tested ApoE-isoform dependent difference in 4F action in the assays of this cell platform. The employed experimental strategy is reported in Fig. 5A (modified from Fig. 3A). On day 4, following the NS replacement with LDS and the application of the ApoE isoforms, the fibroblasts also received 4F lipopeptide or 4F scramble (4F-Sc) as control. After one day of incubation, endogenous cholesterol levels and cell viability were measured. 4F but not 4F-Sc lowered the intracellular cholesterol of LDS/NPC1(-) cells receiving ApoE4 (Fig. 5B). Neither 4F–4F-Sc showed any effect in LDS/NPC1(-) cells receiving ApoE2 or ApoE3. Importantly, the application of 4F to LDS/NPC1(-) in the absence of recombinant ApoEs did not significantly reduce the intracellular cholesterol concentration (Fig. 5C). 4F addition in combination with ApoE4, reduced the BACE1 elevation induced by the disruption of cholesterol trafficking in LDS/NPC1(-) fibroblasts (Fig. 5D, E). This effect was not observed when 4F-Sc was co-administered with ApoE4. Notably, the addition of ApoE2 and ApoE3 alone was sufficient to recover BACE1 intracellular levels (Fig. 5D, E). The normalization of BACE levels clearly indicates a homeostatic influence of the entire amyloid cleavage system of APP, probably of very high significance in pathogenesis. The upstream effect on the NPC inhibition on increased APP and BACE levels clearly supports this interpretation, of lipid dyshomeostasis driving increased likelihood of amyloidgenesis. Next, the effect on cellular viability determined by the MTT assay showed that the 4F lipopeptide, but not 4F-Sc, was able to increase the viability of LDS/NPC1(-) fibroblasts receiving ApoE4 to the level of ApoE2 and ApoE3 receiving cells (Fig. 5E). 4F had no effect on the viability of LDS/NPC1(-) cells receiving ApoE2 and ApoE3 (Fig. 5F). Administering 4F or 4F-Sc alone to NS/NPC1(-) and LDS/NPC1(-) fibroblasts, without the addition of ApoE, did not increase cell viability (Fig. 5G).

**Fig. 6:**
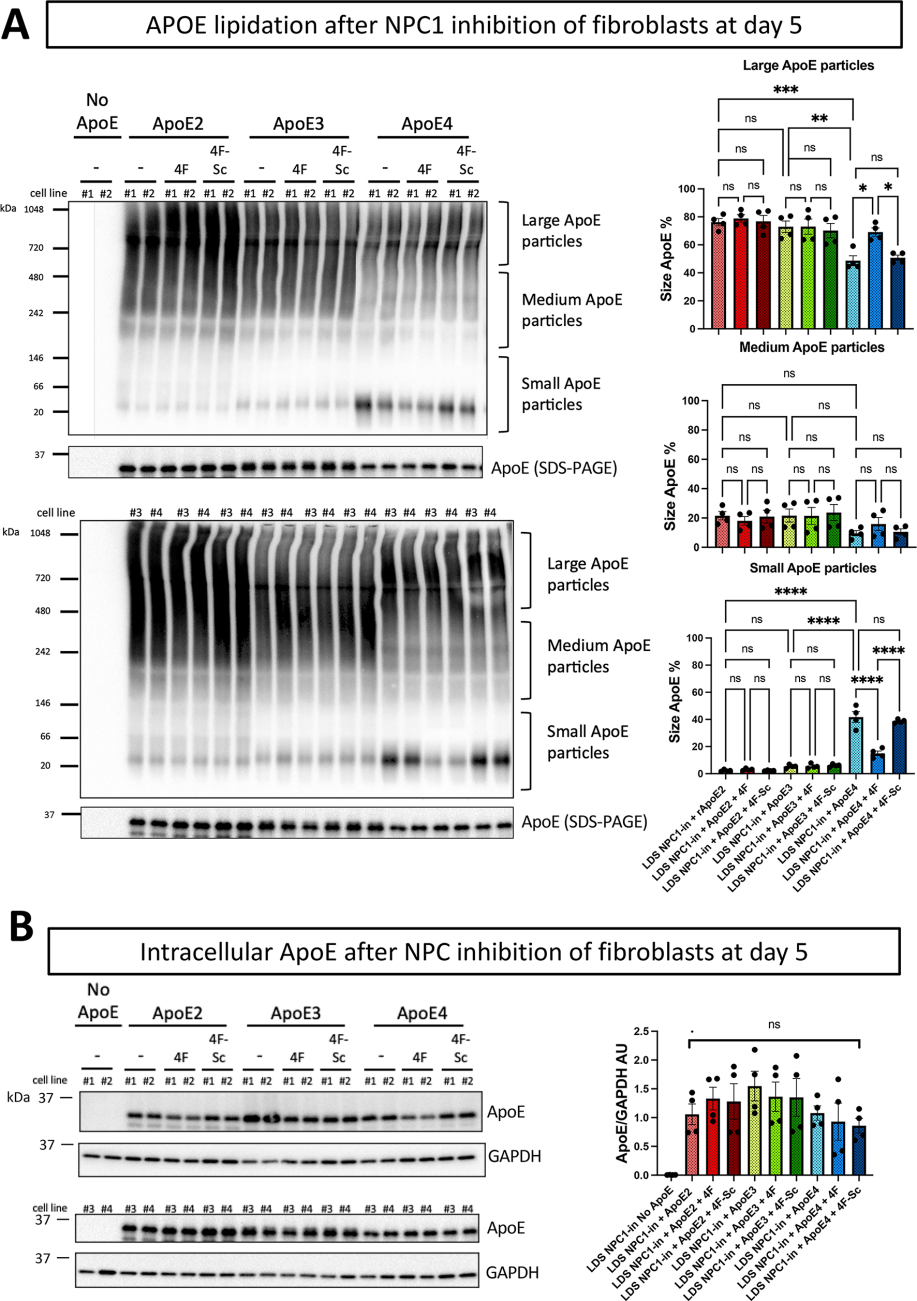
4F enhanced the lipidation of recombinant ApoE4 in NPC1-Inhibited Fibroblasts. (**A**) Supernatants from four fibroblast lines treated with NPC1 inhibitor (NPC1-in) in medium containing lipoprotein-depleted serum (LDS) and receiving recombinant ApoE2, ApoE3, ApoE4 or no ApoE, in the presence or in the absence of 4F or scramble 4F (4F-Sc) peptide (See schematic in Fig. 5A for the experimental design) were subjected to Native-PAGE and SDS-PAGE followed WB and ApoE detection. Left panel: representative Native-PAGE and SDS-PAGE images. Right panel: Quantification of large, medium, and small ApoE lipoparticles following Native-PAGE. The intensity of each grouped band was normalized to represent each lipoprotein species as a percentage of the total band intensity for combined large, medium, and small lipoparticles. (**B**) SDS-PAGE and WB of cell lysates followed by ApoE detection. GAPDH was used as loading control. Left panel: representative WB image. Right panel: relative quantification of ApoE signal normalized over GAPDH. Each dot represents one cell line, and the values are the mean ± SEM of two independent experiments. Pairwise one way ANOVA with post-hoc Tukey for multiple comparison (*p < 0.05, **p < 0.01, ****p < 0.0001). Veh: vehicle (PBS). NPC1-in: NPC1 inhibitor (U18666A).

To understand the mechanism and differentiate between the ApoE isoforms in lipid carrying capacity, the apolipoprotein lipidation was evaluated in the NPC1(-) cell supernatant by Native-Page (Fig. 6), and ApoE was quantified as big, medium, and small particle sizes (Fig. 6A). Notably, ApoE2 was predominantly found in big size particles, ApoE4 in small size particles, and ApoE3 mostly in medium size particles. Interestingly, in the presence of 4F (but not 4F-Sc), the percentage of big size ApoE4 particles increased, primarily driven by a decrease in the percentage of small ApoE4 particles. However, no significant effect on ApoE lipidation was observed when 4F lipopeptide was added to ApoE2 and ApoE3.

To evaluate whether apolipoproteins’ lipidation and their different effects on intracellular cholesterol levels were related to isoform-specific variations in cellular trafficking and uptake, extracellular (Fig. 6A and Supplementary Fig. 5) and intracellular (Fig. 6B) levels of ApoE isoforms were quantified using SDS-PAGE and Western blotting techniques. The analysis revealed no significant differences in the levels of recombinant ApoE within or outside the cells across all experimental conditions. This indicates that the intracellular and extracellular transport of ApoE isoforms does not differ significantly, implying that the variations in the capacity to modify intracellular cholesterol levels are not due to differential trafficking of the ApoE isoforms. In order to simulate the interactions between the key ApoE related enzymatic mechanisms of cholesterol loading we used AlphaFold 3 and provided a in silico based structural analysis of potential interactions between ABCA1 and ApoE4 and the lipopeptide 4F (Supplementary Fig. 6). The simulation provided an understanding of potential interaction sites between ApoE4 and ABCA1 that can be influenced by the lipopeptide. These data are consistent with previous radioactive ligand experiments that have clearly shown that 4F and related peptides can directly interact with ApoA-I and ABCA1^67^, consistent with our new AlphaFold data.

### Validation of the new NPC1 inhibited human fibroblast platform for cholesterol, lipid and APP pathophysiology by using human astrocytes derived from iPSCs

To corroborate and demonstrate the general relevance of the results obtained using human fibroblasts in this cellular platform, astrocytes derived from human induced pluripotent stem cells (hiPSC) were used (see paradigm in Fig. 5A). Astrocytes represent the main source of brain cholesterol, which is then transferred to neurons through an apolipoprotein mediated transport, in particular by ApoE carrier lipid system.

**Fig. 7:**
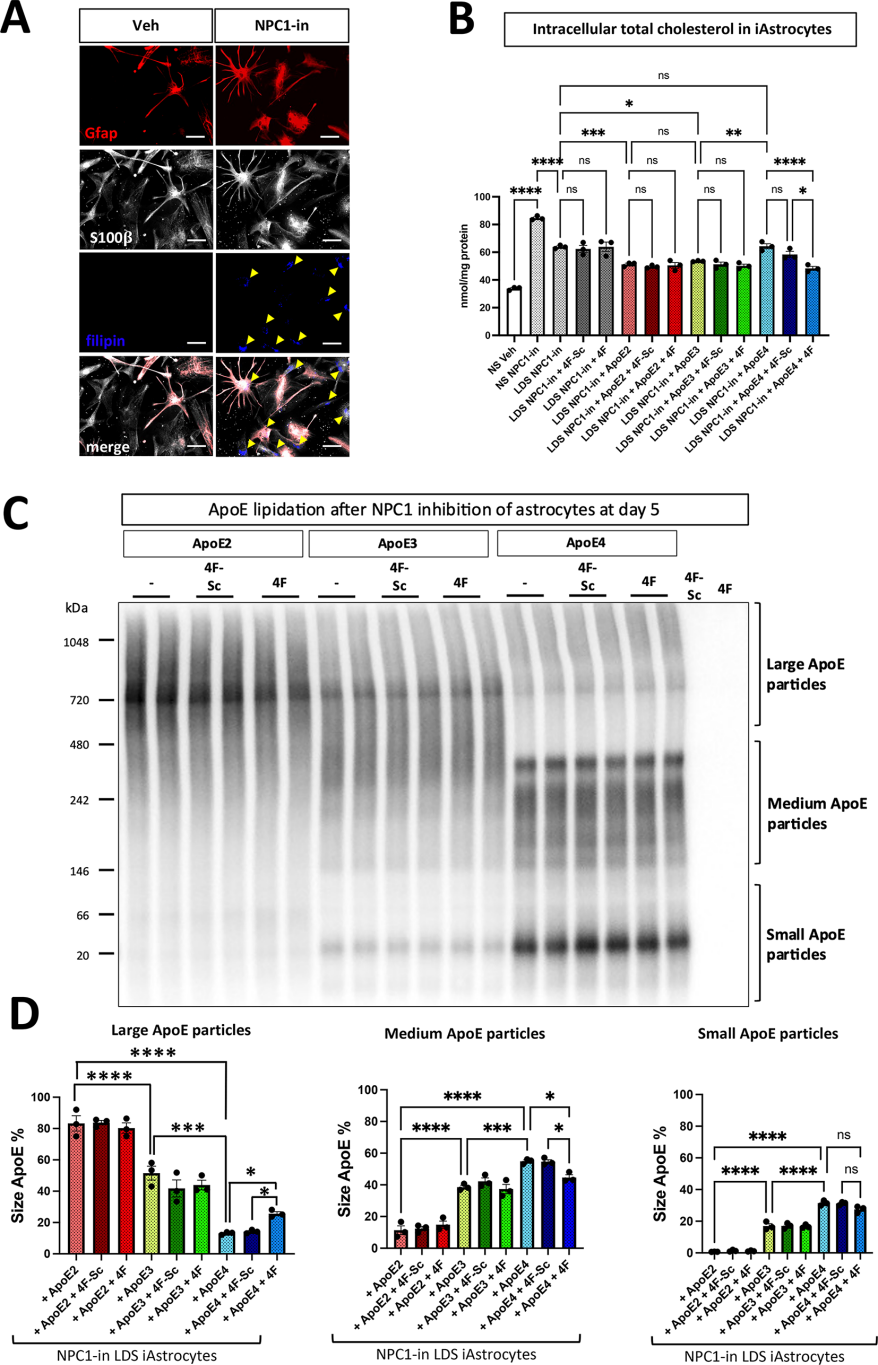

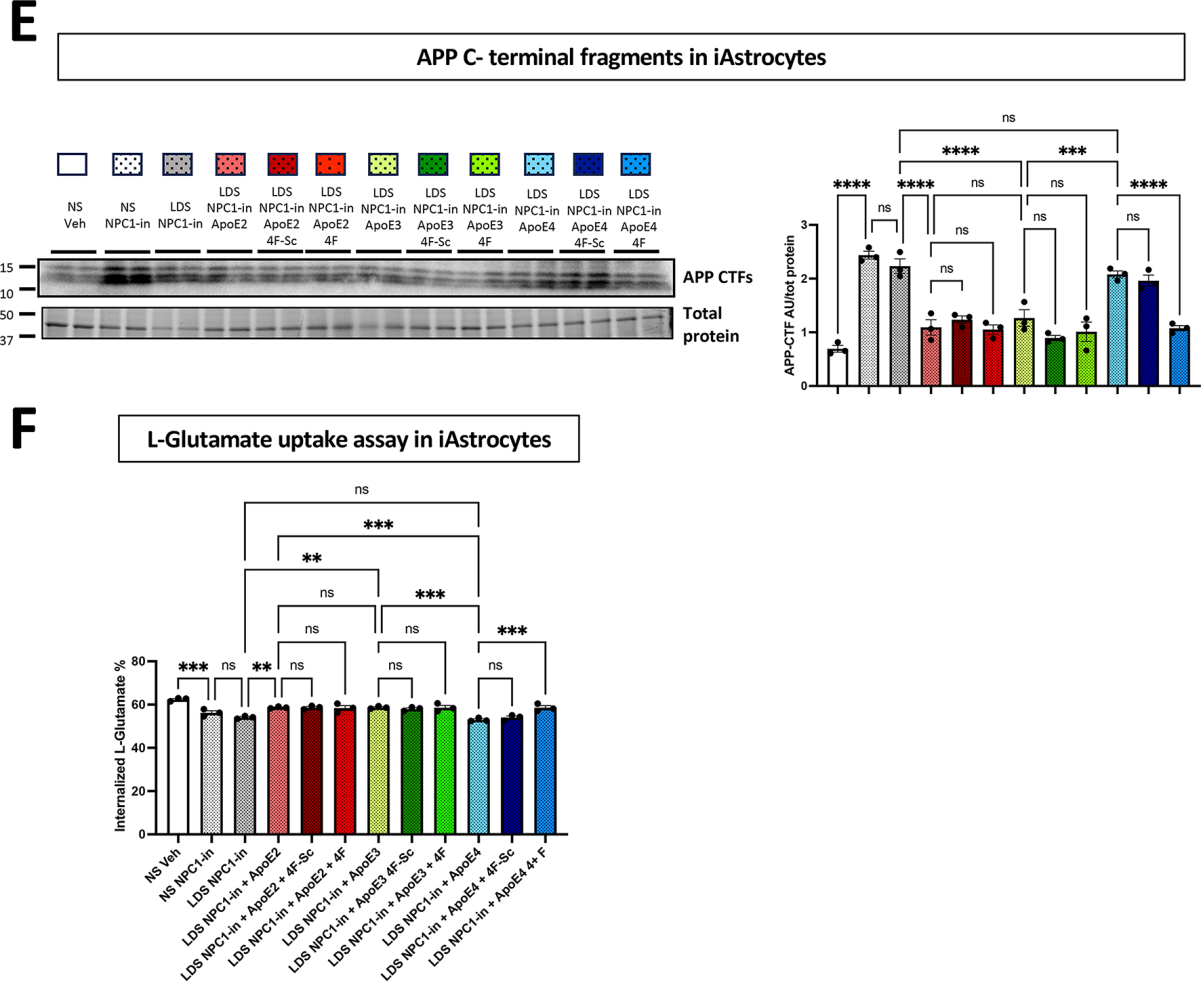
Validation of this cellular lipid challenge platform using hiPSC-derived Astrocytes (iAstrocytes) for cholesterol, functional studies and APP related markers. The experimental design of Fig. 5 was adapted to iAstrocytes to study the effects of recombinant ApoE2, ApoE3 and ApoE4 as well as the 4F lipopeptide, on cholesterol accumulation and functional activity in NPC1-inhibited conditions. (**A**) Representative fluorescence microscopy images illustrating filipin (blue) staining for free cholesterol, GFAP (red), and S100β (grey). Images were acquired at ×40 magnification; scale bar: 50 μm. Yellow arrowheads indicate filipin-stained free cholesterol accumulation in NPC1-inhibited iAstrocytes. 2 coverslips per condition with 10 images acquired from each coverslip, from 2 independent experiments were analyzed. (**B**) Quantitative assessment of intracellular cholesterol levels. (**C**) Representative WB image of ApoE following Native-PAGE analysis of iAstrocyte supernatants. (**D**) Quantification of large, medium, and small ApoE lipoparticles after Native-PAGE. Band intensity for each size category was normalized and presented as percentage of the total intensity for large, medium, and small lipoparticles combined. (**E**) Representative WB images and relative quantification of amyloid precursor protein (APP) C-terminal fragments (CTFs). Proteins detected by WB were normalized to total protein levels at the 50-37 kDa molecular weight range. (**F**) L-Glutamate uptake phenotypic assay. Internalized L-Glutamate in iAstrocytes is expressed as a percentage of the total administered L-Glutamate (100 µM). In B-F, values are the mean ± SEM of three independent experiments. One way ANOVA with post-hoc Tukey for multiple comparison *p < 0.05, **p < 0.01, ***p < 0.001, ****p < 0.0001. Veh: vehicle (PBS). NPC1-in: NPC1 inhibitor (U18666A).

The chemically induced deficiency of NPC1 in astrocytes (here ApoE3/3) resulted in a significant accumulation of cholesterol (NS/NPC1-in vs. NS/Veh) (Fig. 7A, B). Replacing NS with LDS slightly but significantly reduced the lipid accumulation of NPC1(-) astrocytes. The addition of recombinant ApoE2 and ApoE3 (10 $\:{\upmu\:}$g/mL) further lowered the accumulation of cholesterol, while astrocytes receiving the same amount of ApoE4 exhibited higher levels of accumulated cholesterol. Importantly, the addition of the 4F lipopeptide, and not 4F-Sc, improved the cholesterol levels accumulated in the NPC1(-) astrocytes incubated with ApoE4 (Fig. 7B). Additionally, different lipidation levels of ApoE isoforms were found in the supernatant of NPC1(-) astrocytes with ApoE2 presenting mostly in big size particles and ApoE4 in small size particles (Fig. 7C, D). Notably, in the presence of 4F lipopeptide, the percentage of big ApoE4 particles were increased and the percentage of small ApoE particles reduced (Fig. 7D). As in fibroblasts, NPC1(-) hiPSC-derived astrocytes presented higher level of APP C-terminal fragments (CTF) (Fig. 7E). The NS substitution with LDS lowered APP-CTF levels which were further reduced by the addition of ApoE2 and ApoE3. In NPC1(-) astrocytes receiving ApoE4, the levels of APP-CTF remained high. The APP-CTF bands were very clear, but could represent either αCTF or βCTF products, and could also be influenced by phosphorylation states of the CTFs. Importantly, the application of 4F lipopeptide lowered the APP-CTF levels in NPC1 inhibited fibroblasts receiving ApoE4, while no additional effects was observed in ApoE2 and ApoE3 receiving cells. A well-known function of astrocytes in brain physiology is their ability for significant L-Glutamate uptake in the extracellular space^68^. To further evaluate the functional consequences of NPC1 inhibition and lipid load in human astrocytes, their ability to uptake extracellular L-Glutamate was investigated. NPC1 inhibition in astrocytes cultured with FBS (NS) slightly but significantly reduced their Glutamate uptake function (10% $\:\pm\:$3.7 *p* < 0.001 reduction in NS/NPC1(-) condition compared to NS Veh, Fig. 7F). In the LDS/NPC1(-) condition, a 13% $\:\pm\:$3.6 *p* < 0.0001 reduction of L-Glutamate uptake function was observed. The addition of ApoE2 and ApoE3 restored the L-Glutamate uptake function of NPC1(-) astrocytes to the levels of NS/Veh condition, which did not occur when ApoE4 was administered, confirming the ApoE4 as a loss of function isoform also in this physiological context. Importantly, the administration of 4F lipopeptide together with ApoE4, resulted in the recovery of L-Glutamate uptake function in NPC1(-) astrocytes at the same level of ApoE2 and ApoE4 astrocytes. No additional effect on L-Glutamate uptake was observed when 4F was co-administered with ApoE2 and ApoE3.

## Discussion

The inspiration for these in vitro studies come from the realization of the high genetic impact of ApoE4 isoform in Lewy Body Dementia^3,5,25^, Alzheimer Disease^4,7,69,70^ and NPC1 lipid cholesterol intracellular regulation such as seen in Niemann Pick Type C disease^33–37^. Determining lipid homeostasis is critical to understanding the complexities of age-related diseases, such as neurodegenerative disorders. Human genetic variants, particularly the *APOE* ε4 allele, contribute to the onset and progression of neurodegenerative diseases by altering lipid metabolism and organelle function^3–5,56^. There is a reasonable case to be made for a central hypothesis where lipid dysregulation through specific genes related to lipid homeostasis, transport and metabolism are the main drivers of these neurodegenerative diseases. In a broader perspective, including other related age-dependent neurodegenerative diseases, many key etiological and genetic drivers involve lipids such as glycolipids, sphingolipids and their respective functional networks^1,2,24,26,30,65,71^.

### Modeling lipid dysregulation by inhibiting the NPC1 transporter and investigating cellular compensatory mechanisms

The human cellular platform reported here used fibroblasts or astrocytes to evaluate the effects of increased cholesterol levels inside these cells. This was accomplished by blocking the intracellular cholesterol trafficking at the endo-lysosomal level. This experimental set up also allowed examination of how exogenous recombinant ApoE could compensate for lipid transport disruption and intracellular lipids accumulation.

Specifically, the U18666A inhibition of the endo-lysosomal cholesterol transporter NPC1 disrupted cholesterol trafficking towards the endoplasmic reticulum (ER)^43^ and caused significant intracellular accumulation of cholesterol. Since the molecular machinery for cholesterol sensing is localized at the ER^44^, the loss of NPC1 function in our experiments caused a cholesterol mis-localization and mis-sensing, which could explain the paradoxical changes of increased HMGCR and decreased ABCA1, despite the cellular cholesterol overload. These findings agree with previous work where human NPC1 mutant fibroblasts showed lower mRNA and protein expression of ABCA1^19^. As seen in the present in vitro study, the increase in cholesterol synthesis rate was previously reported in a NPC1 mutant model in several organs and tissues^72^. These studies collectively confirm the undesirable feed-forward loop change in cholesterol metabolism; where NPC1 defective cells lose the ability to egress the sterol, but increase the synthesis of it^19,72^. Interestingly, strategies aimed at prompting mobilization of cholesterol to the ER, such as 2-hydroxypropyl-β-cyclodextrin (HPβCD), have been reported to normalize the levels of ABCA1 and HMGCR^73^.

NPC1 inhibition also elevated the intracellular levels of lipid droplets and triglycerides, indicating widespread lipid dysregulation and subsequent detrimental effects on cell viability. Notably, we observed an increase in the levels of the ACSL1 enzyme, responsible for triglyceride synthesis, following NPC1 inhibition. This suggests a broad impairment in the lipid regulatory network due to the mis-localization of intracellular lipids. Our previous findings in vivo demonstrated that endo-lysosomal accumulation of glycolipids in mouse brain, induced by CBE injections, led to elevated levels of NPC1 and ApoE, pointing to a potential link between glycolipid and cholesterol metabolism and trafficking^25^. Taken together these data show a tightly integrated network of lipid regulation, whereby disruption in one pathway could precipitate cascading effects throughout other interconnected pathways.

### Connecting lipid dysregulation with APP metabolism

Recently, *ACSL1* expression was found elevated in postmortem prefrontal cortex microglia of AD patients, particularly in those with the *APOE4* allele^74^. These ACSL1-positive microglia were rich in lipid droplets and were found near beta-amyloid plaques in the AD brain. Perhaps of relevance to increased amyloid formation, in the present study cholesterol and neutral lipids accumulation were associated with elevated APP levels and C-terminal fragments, demonstrating that cholesterol and lipid imbalance directly influence APP metabolism^26,54,55,60,75^. These APP metabolism effects we found, were consistent across human fibroblasts and astrocytes. While the glial APP expression levels are relatively low compared to neurons, the changes observed in APP processing can provide important insight into general and broader cellular stress responses to lipid and cholesterol accumulation in human cell platforms. This indicates general or shared molecular mechanisms, reinforcing the utility of the fibroblast and astrocyte cellular models in studying molecular mechanisms of neurodegenerative diseases. One potential limitation of using fibroblasts and astrocytes is the very low levels of $\:{\upbeta\:}$ -amyloid peptides generated from these cell types. However, these cell types do reveal APP related changes. The current experiments revealed that BACE1 levels increased following NPC1 inhibition—a result suggesting enhanced amyloidogenic processing under conditions of cholesterol and lipid disruption. This cellular assay can also be employed for studying human iPSC derived neurons, astrocytes, microglia and oligodendrocytes using genetic tools and relevant cell type specific markers^76^. A main finding in lipid dysregulated cells due to NPC1 inhibition is that APP protein directly interacted with lipid droplets, an event that possibly contributed to the altered intracellular APP levels and processing. The interaction between cholesterol and APP proteins has been previously reported^75,77^. In fact, APP presents a characterized cholesterol binding site and, when bound to cholesterol, the $\:{\upgamma\:}$-secretase pathway is favored, which increases APP cutting^77,78^. To the best of our knowledge this is the first time that APP and lipid droplets have been demonstrated to colocalize, although multiple lipids have been associated with $\:{\upbeta\:}$ -amyloid plaques such as cholesterol and fatty acids^79–82^.

### Using a human cellular assay to delineate ApoE isoform-specific actions on lipid dysregulation and cell viability

The role of human ApoE isoforms in modulating cellular responses to cholesterol and lipid metabolism alterations was investigated in this five-day cellular platform. In the present study using this cellular platform, equimolar concentrations of each ApoE isoform were applied to lipid accumulating fibroblasts and astrocytes, which allowed the controlled examination of specific ApoE protein function. The impact of specific ApoE isoforms (ApoE2, ApoE3, ApoE4) on several cellular biological variables and cell death demonstrated that recombinant human ApoE isoforms differ in their capacity to manage and influence cholesterol transport, APP processing, cellular function, and cell viability. While ApoE2 and ApoE3 showed compensatory effects in restoring cellular lipid equilibrium with NPC1 inhibition, ApoE4 lacked this restorative potential. The decreased capacity of recombinant ApoE4 on cholesterol efflux was associated with lower lipidation, as previously shown^9,13,65,83^. It has been inferred that this difference in lipo-particle lipid composition in part is due to the lateral chain of Arg112 residue of ApoE4 (instead of Cys112 of ApoE2 and ApoE3). This alters the ApoE4 protein conformation, conferring lower preference for high density lipoprotein formation and higher preference towards very low-, or low-density-lipoproteins^9^. Structural differences between ApoE4 vs. ApoE2 and ApoE3 have also been postulated to impact protein stability, with ApoE4 appearing less stable and prone to degradation^9,10,84^.

### Leveraging the cellular platform to explore strategies to enhance ApoE4 lipidation and function

Using this cellular screening platform to test ApoE4 potentiating drugs, the amphipathic apolipoprotein A-I mimetic peptide 4F was able to reverse the cellular changes associated with cholesterol accumulation, including cell death. The 4F lipopeptide, mimicking the class A amphipathic helices of HDL-associated apolipoproteins, including ApoA-I and ApoE, has been previously reported to facilitate the lipidation of ApoE in an ABCA1-dependent manner^66^. The current study demonstrated in vitro that the 4F lipopeptide combined with recombinant human ApoE4, reduced cellular lipid burden, lowered APP C-terminal fragments and BACE1 levels, increased ApoE4 lipidation, and enhanced cellular functions and viability. This proof-of-concept study provides an opportunity to biologically optimize ApoE4 function in neurodegenerative diseases. The exact mechanism by which 4F restored ApoE4 lipidation remains to be elucidated. Potential mechanisms may include: (i) 4F interaction with ApoE4, enhancing its affinity for cholesterol; (ii) 4F stabilization of ABCA1 on the plasma membrane, leading to increased cholesterol efflux; or (iii) 4F promotion of ABCA1-ApoE4 interaction, which would facilitate a greater cholesterol load on ApoE4. The protein simulation by AlphaFold (see Suppl. Figure 4) suggested that the synthetic 4F lipoprotein is potentially able to interact with both ABCA1 and ApoE4 and supports previous investigations directly linking ABCA1 and ApoE/ ApoA-I structural interactions. Relevant molecular enhancers in this system are likely to provide enhanced cholesterol management by the APOE4 system and thereby reducing its potential toxic effects in its less lipidated form (see Fig. 3). This strength of our data is the design of the experiments to specifically to test equimolar doses of added ApoE2, 3 and 4 under the condition of NPC1 inhibition. Under those platform and experimental conditions, and with different human cell types, our results in this platform are clear, and support an optimal functional effect by ApoE2 and ApoE3 by themselves, and a beneficial cholesterol efflux, APP and BACE1 normalization as well as cell viability in the presence of ApoE4 with 4F peptide.

Overall, these results shed new light on the profound and detrimental feed-forward loop initiated by intracellular cholesterol trafficking impairment through NPC1 inhibition. This in vitro model captured the dynamic cascade of cellular dysfunction that arises from lipid dysregulation, leading to elevated intracellular lipid levels, and also increased APP levels and altered processing. This biological response shows that the APP molecular system is linked with lipid stress. The individual APP-related enzymes involved, including BACE1, changed homeostatically in the protein levels measured. Presumably this also influences their total enzyme activity. The addition of 4F (known to interact with ABCA1 and increase lipid load of ApoE) caused improved cholesterol efflux but also normalized APP levels and APP processing enzymes. In conclusion, the human cellular platform developed in this study allowed us to evaluate the ApoE4 isoform in lipid dysregulation scenarios and underscores the potential for therapeutic interventions targeting the lipidation of ApoE4. These strategies could have a significant impact in treating and managing neurodegenerative diseases characterized by lipid imbalance.

## Methods

### Cell cultures

Four lines of healthy subject (HS) derived fibroblasts were purchased from the Coriell Institute for Medical Research (#AG11489, #AG11743, #AG05265, #AG04461 named HS21, HS23, HS22 and HS28 respectively). All the lines were assessed to be mycoplasma free. The passage numbers for all fibroblast lines used ranged from 10 to 19. Cells were cultured onto 6-mw, 24-mw (with coverslips) or 48-mw at 10000 cells/cm^2^ cell density in cell medium composed of DMEM, High Glucose (Thermo Scientific #11965092), 10% fetal bovine serum (FBS, Sigma-Aldrich #12306 C), 1% Penicillin-Streptomycin (10,000 U/mL, Thermo Scientific #15140122), 0.5% L-Glutamine (Gibco #25030-081), 1% MEM Non Essential Amino Acids Thermo Fisher Scientific #11-140-050) at 37∘C, 5% CO_2_.

A commercial line of hiPSC derived astrocytes (iCell Astrocytes) was purchased by FUJIFILM Cellular Dynamics, Inc. (FCDI) (# R1092). The cells were seeded onto laminin pre-coated (10 µg/ml Sigma-Aldrich #L2020) 6-mw, 24-mw (with coverslips) or 48-mw plates at a density of 50,000 cells/cm^2^ in cell medium composed of DMEM/F-12, HEPES (Thermo Fisher Scientific #11330), 2% FBS and 1% N2 Supplement (Thermo Fisher Scientific #17502048) at 37∘C, 5% CO_2_. The genotype of iCell Astrocytes (APOE3/3) was provided by FCDI. The same batch of astrocytes was used across experimental conditions, ensuring consistency and minimizing any potential variability due to cell batch variability or heterogeneity.

### Evaluation of ApoE genotype

Genomic DNA was extracted from human fibroblast lines utilizing the DNeasy Blood & Tissue Kit (Qiagen, #69504) as per the manufacturer’s protocol. Amplification of the ApoE gene was performed using specific primers: forward primer 5’-CAGGTCACCCAGGAACTGAG-3’ and reverse primer 5’-CACCTGCTCCTTCACCTCG-3’. The polymerase chain reaction (PCR) entailed 30 cycles with Platinum Taq DNA Polymerase (Thermo Scientific, #10966018) and the following thermal cycling conditions: denaturation at 94°C for 1 minute, annealing at 57°C for 2 minutes, and extension at 72°C for 3 minutes. The resultant PCR products were forwarded to Eton Bioscience (Boston, USA) for sequencing of the ApoE locus, employing an additional set of primers: forward primer 5’-GCCTACAAATCGGAACTGGA-3’ and reverse primer 5’-CTGCCCATCTCCTCCATC-3’.

### RNA extraction and quantitative PCR (qPCR)

Total RNA was isolated from fibroblasts and human-induced pluripotent stem cell (hiPSC)-derived astrocytes using the RNeasy Mini Kit (Qiagen, #74104), with the final elution in 30 µL of RNase-free water. RNA concentration and purity were assessed with the NanoDrop^®^ ND-1000 UV-Vis Spectrophotometer (Thermo Fisher Scientific). To remove any contaminating genomic DNA, samples underwent DNase I treatment (2.73 U/µL) for 15 min at 23 °C. Reverse transcription of 1 µg of total RNA was conducted using the SuperScript III First-Strand Synthesis System and Oligo-dT20 primers (Life Technologies, #18080-051) according to the provided protocol.

Quantitative PCR was carried out in triplicate for each sample using SYBR Green PCR Master Mix (Thermo Fisher Scientific, #4367659). The primer pairs for ApoE were as follows: forward 5′-GTTGCTGGTCACATTCCTGG-3′ and reverse 5′-GCAGGTAATCCCAAAAGCGAC-3′. Hypoxanthine phosphoribosyltransferase (HPRT) served as the endogenous control, with primers forward 5′-CCTGGCGTCGTGATTAGTGAT-3′ and reverse 5′-AGACGTTCAGTCCTGTCCATAA-3′.

Thermocycling was initiated with a 10-minute enzyme activation step at 95 °C, followed by 40 cycles of denaturation for 15 s at 95 °C and annealing/extension for 1 min at 59 °C on the StepOnePlus™ Real-Time PCR System (Applied Biosystems, #4376600). Ct values for ApoE were normalized to HPRT and the relative expression levels were calculated using the comparative Ct (ΔΔCt) method.

### Cell treatments and platform methodology

The day subsequent to cell plating, U18666A (EMD Millipore, #662015) or phosphate-buffered saline (PBS, as vehicle control) was introduced to the cultures at a concentration of 3 µg/mL in complete medium supplemented with normal fetal bovine serum (NS). The cultures were maintained with U18666A for a duration of three days. Following a two-day incubation, the medium was aspirated, and cells were rinsed thrice with Dulbecco’s Modified Eagle Medium/Nutrient Mixture F-12 (DMEM/F-12). Subsequently, cells were provided with fresh culture medium in which NS was substituted with lipoprotein-depleted serum (LDS, Kalen BioMedical #880100), complemented with 10 µg/mL of recombinant human apolipoproteins E^51–53^, produced in Escherichia Coli: ApoE2 (Sigma-Aldrich #SRP4760), ApoE3 (Sigma-Aldrich #SRP4696), or ApoE4 (Sigma-Aldrich #A3234). ApoE proteins derived from E. coli typically exhibit lower lipidation levels compared to those isolated from plasma or synthesized in eukaryotic cells^51–53^. The application of poorly lipidated recombinant ApoE to the human NPC1 disrupted cells allowed to evaluate the ability of exogenous human apolipoprotein variants, in the absence of other serum derived lipoproteins, to take up intracellular accumulating lipids and compensate for defective intracellular lipid transport. The endotoxin content in the E. Coli-produced recombinant ApoE isoforms was verified to be less than 0.1 ng/µg by the manufacturer using the Limulus Amebocyte Lysate (LAL) method. This endotoxin level was not sufficient to provoke inflammatory responses in fibroblasts and astrocytes, as evidenced by the absence of differences in IL-6 secretion post-administration (data not shown). Cells were incubated with the recombinant ApoE proteins for one additional day preceding the execution of biological and biochemical assays.

In experiments designed to modulate ApoE functionality, peptides 4F ^({ASP}^{TRP}{PHE}{LYS}{ALA}{PHE}{TYR}{ASP}{LYS}{VAL}{ALA}{GLU}{LYS}{PHE}{LYS}{GLU}{ALA}{PHE}) and a scrambled version of 4F ^({ASP}^{TRP}{PHE}{ALA}{LYS}{ASP}{TYR}{PHE}{LYS}{LYS}{ALA}{PHE}{VAL}{GLU}{GLU}{PHE}{ALA}{LYS}) (GenScript USA, Inc.), or dimethyl sulfoxide (DMSO, as vehicle control), were introduced into the culture medium at a final concentration of 5 µM, a 17.5-fold higher molar ratio than that of the ApoEs, during the last day of incubation^66^.

To assess the influence of U18666A on neutral lipid metabolism, 1 µM Triacsin C (Cayman Chemical Company #10007448) was incorporated into the culture medium. Additionally, to determine U18666A’s impact on macro-autophagy, cultures were treated with either 1 µM Rapamycin (Cayman Chemical Company Cat#13346) or 200 nM Bafilomycin A1 (Sigma-Aldrich #SML1661). Bafilomycin A1 was administered in the final 4 h of cell incubation.

### ApoE ELISA

Media collected from cells under the different experimental conditions were frozen at -80∘C. The day of the assay, media were thawed on ice and diluted twice in 1X assay diluent buffer before the evaluation with human ApoE Elisa kit (Cat# EHAPOE, Invitrogen), following the manufacturer instructions. Colorimetric reactions were measured by SpectraMax M3 microplate reader (Molecular Device) by reading the absorbance at 450 nm.

### Gel electrophoresis and Immunoblotting

Cells were harvested and lysed with cold RIPA buffer (Thermo Fisher, #PI89900) supplemented with Halt protease along with phosphatase inhibitor cocktail and EDTA (Thermo fisher, #78440). Cells were incubated on ice for 30 min after which they were sonicated (BioLogics Inc, Model 150 V) and spun down. Protein concentration of the supernatant was determined using BCA assay (Thermo fisher, #23225). Equal amount of proteins were mixed with Pierce lane marker reducing sample buffer (Thermo fisher, #39000), boiled at 95 ∘C for 5 min, loaded onto precast 4–20% gradient Criterion Tris–HCl protein gels (Bio-Rad, #3450033) and were electrophoresed at 75 V for 10 min followed by 150 V for 1 h. The proteins were transferred onto a PVDF membrane (Bio-Rad, #1704157) using the Trans-blot turbo system (Bio-Rad) at 25 V and 1.3 Amps for 15 min, followed by blocking of the membranes in blocking buffer comprising 1 × Tris-buffered saline (Bio-Rad, #170–6435) with 0.1% Tween 20 (American Bioanalytical, #AB02038-01000) and 5% blotting grade blocker (Bio-Rad, #170–6404). Membranes were then incubated overnight at 4 °C (on a shaker) with the following primary antibodies diluted in blocking buffer: anti-ABCA1 (Abcam # ab7360, 1:1000), anti- Anti-HMGCR (Abcam #ab242315, 1:1000), anti-ACSL1 (Proteintech, #13989-1-AP0 1:1000), anti-beta Amyloid (Thermo Fiscer Scientific #CT695, 1:500), anti-BACE1 (Cell Signaling Technology, #5606S, 1:1000), anti-p62 Cell Signaling Technology #5114, 1:1000) and anti-LC3 (Millipore #ABC232, 1:1000). The membranes were washed 4 times (10 min incubation on the shaker at room temperature) in TBST (1 × Tris-buffered saline (Bio-Rad, #170–6435) with 0.1% Tween 20 (American Bioanalytical, #AB02038-01000) after which they were incubated in appropriate HRP conjugated secondary antibodies (1:10000) diluted in blocking buffer, for 1 h at room temperature (on the shaker). Following another 4 washes with TBST (10 min incubations on the shaker at room temperature), the signals were developed using Advansta WesternBright Sirius chemiluminescent substrate (Advansta, K-12043-D20) or SuperSignal West Pico Plus chemiluminescent substrate (Thermo fisher, #34579), and imaged using Chemidoc XRS with Image Lab software. Densitometry analysis was performed using Bio-Rad Image Lab software, and target protein bands were normalized to stain-free total protein bands. Stain-free total protein bands were obtained by exposing the PVDF membrane to UV light after protein transfer. For normalization, bands within the 50 − 37 kDa molecular weight range were selected. Densitometry analysis was performed using ImageJ software and all protein bands were normalized over stain-free total protein levels. Original, uncropped images of immunoblots are provided in Supplemental Figs. 7–12.

Non-denaturing gradient gel electrophoresis (NDGGE) was used to assess ApoE lipidation in the cell culture media. Fresh media were run on 4–20% polyacrylamide tris-glycine gels in the absence of sodium dodecyl sulfate, reducing agents or sample boiling, at 100 V for 1 h. Native protein ladder (NativeMark Unstained Protein Standard, #LC0725, ThermoFisher) was loaded to track protein molecular weights. Proteins were transferred onto PVDF membranes at 100 V for 90 min and probed for ApoE (anti-ApoE, Millipore #178479, 1:500), followed by horseradish peroxidase-conjugated secondary antibody and chemiluminescence detection using enhanced chemiluminescence reagents. The intensity of ApoE lipoprotein bands derived from Native PAGE was quantified using Image Lab Software as follows: The detected ApoE lipoprotein bands were categorized into three groups based on their electrophoretic migration. Bands located at the top of the gel (~ 700 kDa < mw < ~ 1000 kDa) were classified as Large-sized lipoparticles, bands in the middle of the gel (~ 60 kDa < mw < ~ 700 kDa) were classified as Medium-sized lipoparticles, and bands at the bottom of the gel (mw < ~ 60 kDa) were classified as Small-sized lipoparticles. To represent each lipoprotein species in a sample, the value of the specific band intensity was expressed as a percentage of the total intensity of the Large, Medium, and Small lipoprotein bands combined. This allowed for the relative quantification of each lipoprotein species in terms of their contribution to the overall band intensity.

### Cholesterol measurement

Total cholesterol levels and the levels of esterified and non-esterified cholesterol in cell lysates and supernatants were measured by Amplex Red Cholesterol Assay Kit (Thermo Scientific #A12216) following manufacturer instructions. Briefly, 2 µg of cell proteins or 10 µL of cell supernatant, were diluted in 50 µL of Reaction buffer 1X and heated at 60 °C for 20 min. Next, the diluted samples were loaded onto black-clear bottom 96-mw and 50 µL or reaction mixture containing (300 µM Amplex Red reagent, 2U/mL HRP, 2 U/mL cholesterol oxidase, and 0.2 U/mL cholesterol esterase) was added to each well and the plate was incubated at 37∘C for 30 min. At the end of the incubation, the fluorescence was read at ex/em = 560–590 nm. To measure Free cholesterol, cholesterol esterase was omitted in the enzyme reaction mixture. Triplicate of each sample were assayed. The samples were run together with cholesterol standards to build the standard curve and interpolate sample concentrations.

### Fluorescence microscopy

The cells plated onto coverslips were fixed in 4% PFA (in PBS) for 20 min at room temperature. After rinsing the cells three times in PBS, the cells were incubated with blocking/permeabilizing solution [10% normal donkey serum (Jackson ImmunoResearch Laboratories #017-000-121) in PBS-T (0.1% triton-X100 in PBS)] for 30 min, at room temperature (RT) under mild shaking. Following, the incubation with primary antibodies diluted in blocking/permeabilizing solutions was carried out for 2 h at RT or overnight at 4 °C, under mild shacking. The primary antibodies and dilutions were the following: LAMP1 (Abcam #ab25630, 1:15), APP (Thermo Fisher Scientific #CT695, 1:200), VIMENTIN (Sigma-Aldrich #V4630, 1:500), GFAP (Synaptic System #173 004, 1:500), S100β (Abcam #ab52642, 1:200). After primary antibody incubation, the cells were washed three times in PBS and incubated 2 h at RT, under mild shacking, with Alexa-Fluor conjugated secondary antibodies (Invitrogen) diluted 1:500 in PBS. For the staining of free cholesterol, filipin reagent (Sigma-Aldrich #F9765) was added to the mixture of secondary antibody mixture in PBS, at the concentration of 0.1 mg/mL. For the staining of neutral lipids, BODIPY^®^ 493/503 (Thermo Fisher Scientific #D3922) was added to the secondary antibody mixture in PBS at the final concentration of 10 µg/mL. At the end of the incubation, the cells were washed 3 times in PBS and nuclei were stained with Hoechst 33,342 (Thermo Scientific #H3570) 1 µg/mL in PBS, 10 min at RT. Nuclei staining was omitted during filipin staining. Coveslips were mounted on slides in ProLong Diamond medium (Thermo Scientific #P36970).

Fibroblasts images were acquired at 40X or 100X using a Leica TCS-SP8 confocal microscope, with a stack height of 0.5 μm and equipped with the LAS-X software. HiPSC-derived astrocytes images were acquired with the BZ-X800LE inverted Keyence fluorescence microscope, at 40X magnification. For colocalization analysis of APP + and bodipy + signals, confocal stack images were analyzed with a Matlab implementation of the Costes algorithm^85^ and Pearson’s correlation coefficient is depicted on the graph.

### Viability assays

Fibroblasts viability was determined by MTT (Thermo Scientific #M6494) and CyQUANT™ Cell Proliferation (Invitrogen #C7026) assays. Both assays were performed in 96-mw. MTT assay was used to assess the cell metabolic activity. Briefly, the MTT stock solution (4 mg/ml in PBS) was diluted in cell culture at the concentration of 2.4 mM for 4 h at 37 °C. Next, cell medium was carefully removed and replaced with 2-propanol: formic acid, 95:5 (v/v). Plates were gently shaken prior to reading the absorbance at 570 nm with a microplate spectrophotometer. CyQUANT™ Cell Proliferation was used to evaluate the cell number upon treatments and the manufacturer instructions were followed.

### Triglycerides measurement

Triglyceride evaluation was performed using Triglyceride Quantification Assay Kit (Abcam #ab65336) following the manufacturer instructions.

### L-Glutamate uptake assay

After treatments, 48-mw plated astrocytes were evaluated for their ability to uptake exogenous L-Glutamate^86^. L-Glutamate (Abcam #ab120049) was dissolved in distilled water at the concentration of 25mM, for 30 min at 37 °C. L-Glutamate stock solution was then diluted in HBSS (plus Ca^2+^ and Mg^2+^) (Life Technologies #14025-092) at 100 µM. Cells were pre-incubated with 100 µL of HBSS (without Ca^2+^ and Mg^2+^) (Thermo Fisher Scientific #14175103) for 30 min. Subsequently HBSS was replaced with 100 µM L-Glutamate solution and the cells were incubated for 4 h in the incubator. Subsequently, cell supernatant was collected, and remaining L-Glutamate was measured with L-Glutamate assay kit (Sigma-Aldrich #MAK004) following the manufacturer instructions. Cells on the plate were lysed in RIPA buffer and proteins were quantified with BCA. The percentage of L-Glutamate internalized by the cells was normalized on the protein content. The assay was run in duplicate from three replicates for each experimental condition.

### In Silico modeling of protein and peptide interactions

The amino acid sequences of human ABCA1 (ENST05220143924.1), APOE (ENST05220078245.1^18^) and 4F (DWFKAFYDKVAEKFKEAF) were imported from the genome browser (Ensembl, https://useast.ensembl.org/index.html) or manually into the cloud-based protein modeling software (AlphaFold, https://alphafoldserver.com, submitted June 2024). The C-R amino acid substitution was incorporated into APOE4 and the N-terminal signal domain of MKVLWAALLVTFLAGCQA was removed from both APOE3 and APOE4 amino acid sequences. Post-translational modifications (PTMs) of APOE and ABCA1 were identified (UNIPROT, P02649 and O95477, respectively and PhosphoSite Plus) and added using parameters in the modeling software that most closely represented the expected native PTM. S-palmitoyl-L-cysteine was added to cysteine at amino acid 112 of APOE3 alone (https://swisspalm.org/proteins/P02649^87,88^). Both APOE3 and APOE4 predicted structures were phosphorylated at serine 129 and 197. Similarly, both APOE3 and APOE4 protein structures were glycosylated at threonine 8, 18, 194, 289, serine 290 and 296 by N-acetyl-beta-D-glucosamine^89^ and acetylated at lysine 242 by N6-acetyl-L-lysine (inferred from^90^). S-palmitoyl-L-cysteine was added to cysteine 3, 23, 1110 and 1111 of ABCA1. Asparagine 14, 98, 151, 161, 196, 244, 292, 337, 349, 400, 478, 489, 521, 820, 1144, 1296, 1453, 1506, 1639, 2046 and 2240 of ABCA1 were glycosylated by N-acetyl-beta-D-glucosamine.

The predicted template modeling (pTM) score and the interface predicted template modeling (ipTM) score were calculated automatically by the software^91^. pTM > 0.5 suggested high similarity of the complex with the protein structures. ipTM > 0.8 represented high quality prediction of protein subunit position. ipTM < 0.6 represented a poorly predicted protein subunit position. Graphical representation of the amino acid conformation was color coded according to confidence of prediction; blue = very high confidence, turquoise = confident, yellow = low confidence, orange = very low confidence.

### Statistical analysis

Statistical data analysis was performed in GraphPad Prism software version 8.4.2. All data are expressed as arithmetic mean ± SEM. Paired two-tailed student’s t-test or One-way ANOVA followed by post hoc testing was used as appropriate and the test used for each analysis is mentioned in the figure legends. In all cases, alpha was set at 0.05 and P value < 0.05 was considered significant for all analyses.

## Electronic supplementary material

Below is the link to the electronic supplementary material.

Supplementary Material 1

## Data Availability

The datasets generated during and/or analysed during the current study are available from the corresponding author(s) on reasonable request. Sequencing data for APOE (as previously described in Gonzales et al., 2021: 10.3390/genes12010004) in the human fibroblast lines used in this study is provided in Supplemental Figure 1.
