# Supplementary material for: ApoE4 requires lipidation enhancement to resolve cellular lipid and protein abnormalities following NPC1 inhibition
Source: Sci Rep. 2025 Apr 29;15:15051. doi: 10.1038/s41598-025-96531-4 (PMC12041514; doi:10.1038/s41598-025-96531-4)
Supplement: Supplementary file 1 — Supplementary Material 1 [file 41598_2025_96531_MOESM1_ESM.pdf]

Supplemental Information for Di Biase E. et al

Title:

ApoE4 requires lipidation enhancement to resolve cellular lipid and protein abnormalities following NPC1 inhibition

Authors

Erika Di Biase, Kyle Connolly, Ingrid Crumpton, Oliver Cooper, Penelope Hallett*, Ole Isacson*.

Affiliation:

Neuroregeneration Institute, McLean Hospital / Harvard Medical School, Belmont MA 02478, USA.

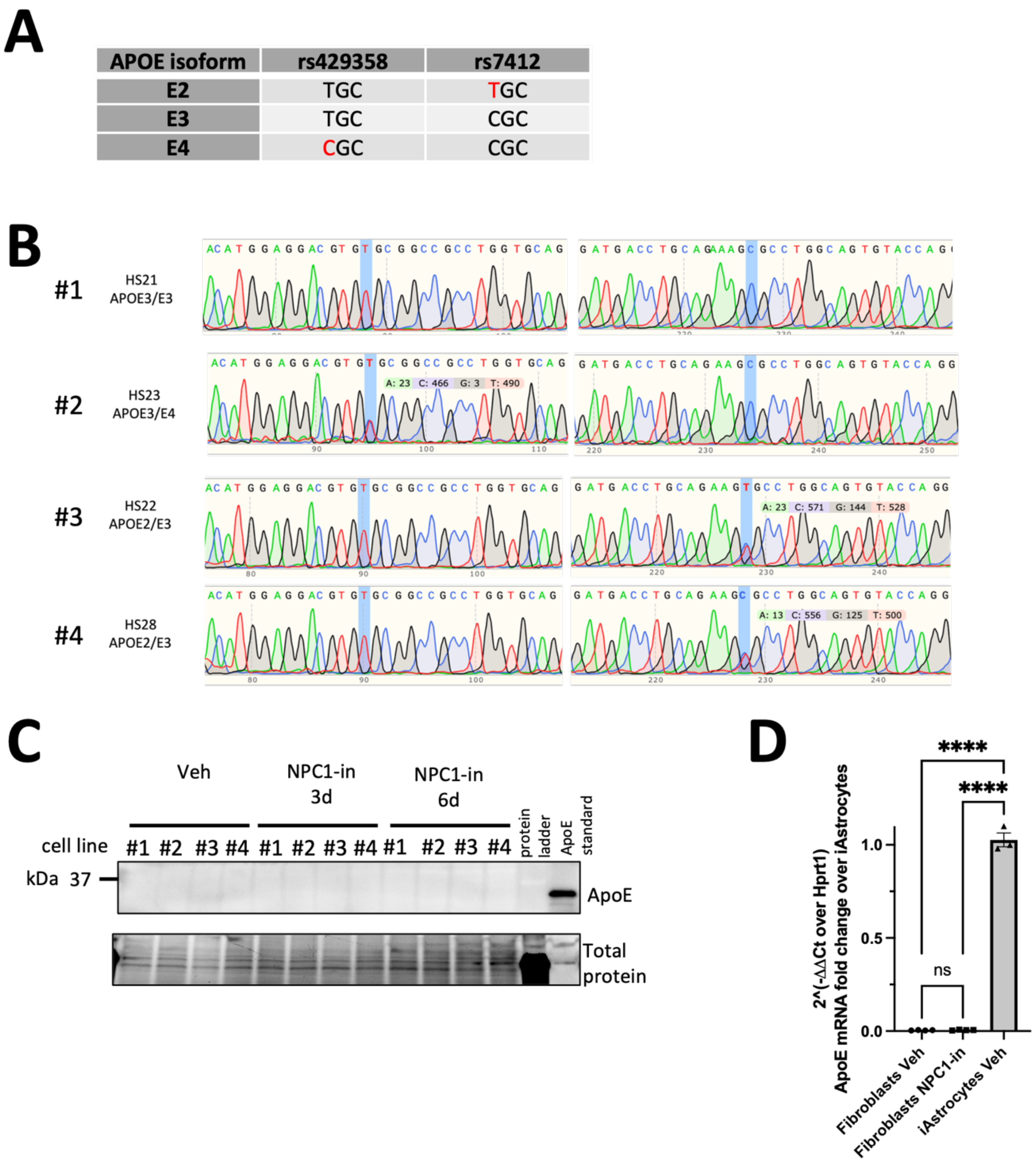

Supplementary Figure 1. Characterization of ApoE genotype and expression in human fibroblast lines. **A)** Single nucleotide polymorphisms (SNPs) that define the three ApoE isoforms are presented: rs429358 (T>C) and rs7412 (C>T). **B)** Identification of the ApoE genotype in the fibroblast lines under investigation. **C)** ApoE WB on four fibroblast lines treated with either PBS (Vehicle, Veh) or 3 µg/mL U18666A for 3 days (NPC1 inhibitor, NPC1-in 3d) and 6 days (NPC1-in 6d). Stain-free gel image depicting total protein content is used as loading control. **D)** The four fibroblast lines were treated with PBS (Vehicle, Veh) or U18666A for 2 days (NPC1-in). ApoE mRNA levels were subsequently assessed by quantitative PCR and compared to those in human induced pluripotent stem cell (hIPSC)-derived astrocytes (iAstrocytes). Values are the mean ± SEM of three (C) or two (D) independent experiments. One way ANOVA with post-hoc Tukey for multiple comparison (****p<0.0001).

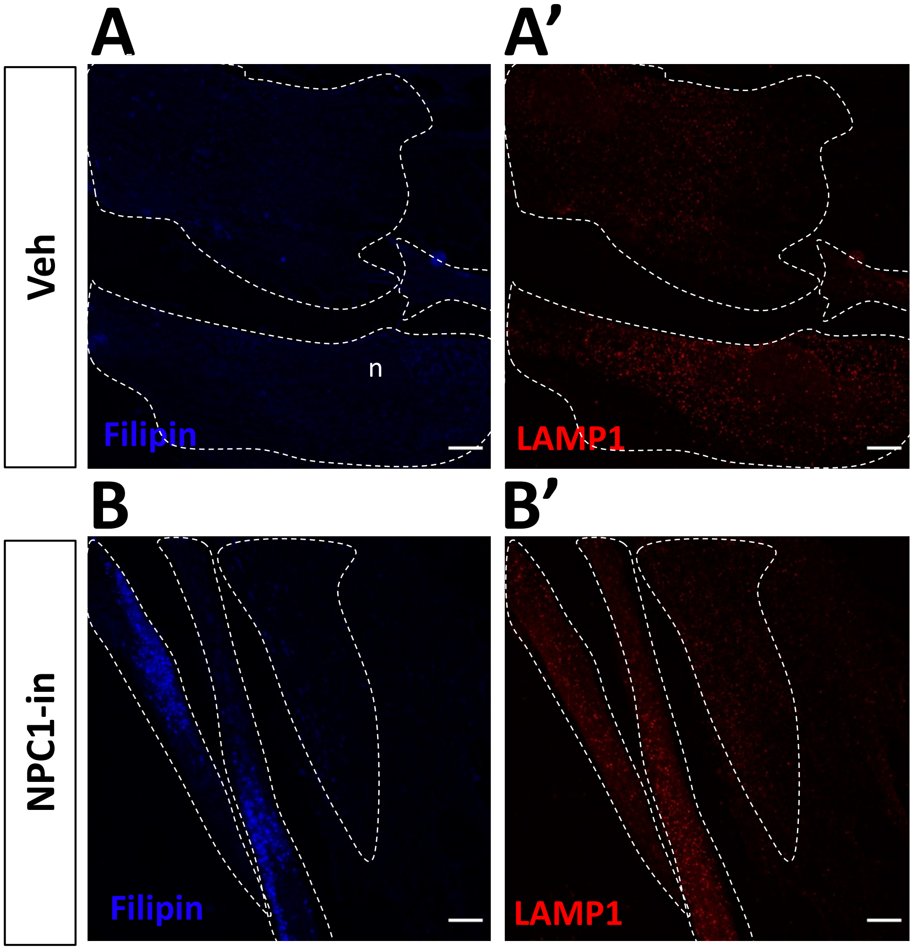

Supplementary Figure 2. Intracellular filipin accumulation following NPC-1 inhibition. Representative single channel confocal images of filipin (blue) **(A, A’)** and LAMP1 (red) **(B, B’)** immunosignals following vehicle or NPC1-inhibition (see Figure 1C for merged images). 100X magnification, scale bar: 10 μm.

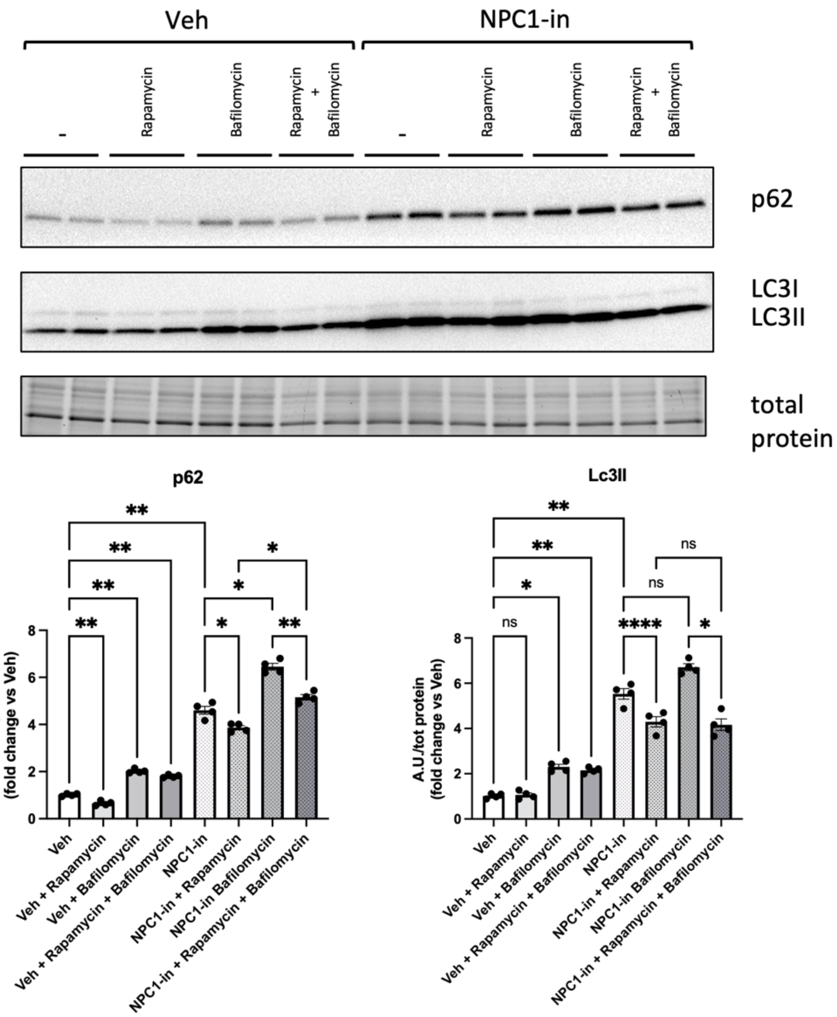

Supplementary Figure 3. Slowdown of macroautophagic flux in NPC1-in fibroblasts. Fibroblasts were treated for two days with either NPC1 inhibitor (NPC1-in; U18666A) or vehicle (Veh, PBS), in the presence of rapamycin and/or bafilomycin, which serve as macroautophagy activator and inhibitor, respectively. Autophagic markers were then evaluated using WB. Top Panel: Representative WB images displaying the levels of p62 and LC3 (I and II) from two fibroblasts lines. Bottom Panel: Quantitative analysis of WB immunosignals for p62 and LC3. It is noteworthy that NPC1-in treatment elevated both p62 and LC3-II levels in a manner similar to bafilomycin treatment, implying a deceleration in macroautophagic flux. Values are the mean ± SEM of two independent experiments using four fibroblasts lines. (N=4). Pairwise one way ANOVA with post-hoc Tukey for multiple comparison (*p<0.05, **p<0.01, ***p<0.001, ****p<0.0001).

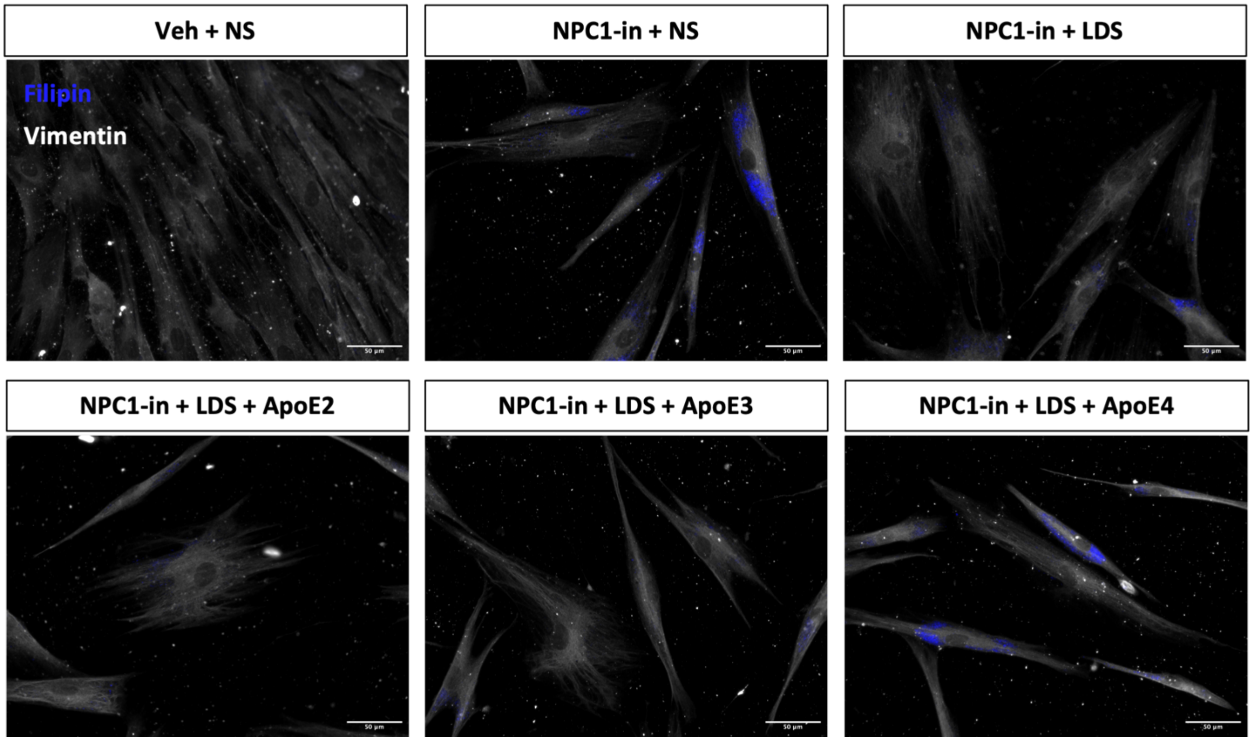

Supplementary Figure 4. Differential effects of ApoE isoforms on free cholesterol levels in NPC1-inhibited fibroblasts shown by ICC instead of biochemistry. Fibroblasts were treated with the NPC1 inhibitor U18666A and cultured for 2 days in medium supplemented with 10% fetal bovine serum (FBS, termed normal serum, NS). The medium was then replaced with lipoprotein-depleted serum (LDS) medium, supplemented with equimolar concentrations of recombinant ApoE isoforms (ApoE2, ApoE3, and ApoE4), and cultured for an additional day (see Figure 3A for the experimental schematic). Immunocytochemistry (ICC) images are presented, showing staining for free cholesterol via filipin probe (blue) and Vimentin (grey). Images were captured at 40X magnification; scale bar: 50 µm.

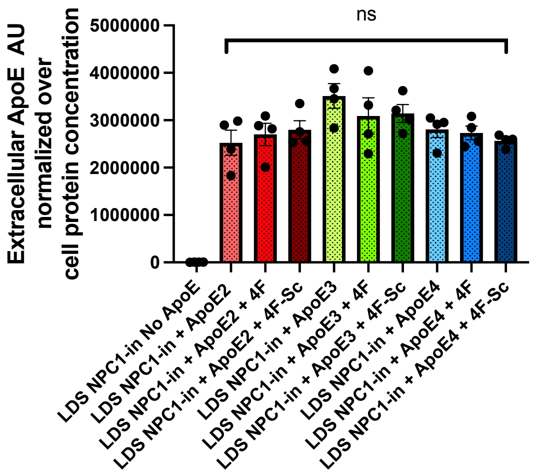

Supplementary Figure 5. Quantification of extracellular ApoE levels in supernatants from four fibroblast lines treated with NPC1 inhibitor (NPC1-in) in medium containing lipoprotein-depleted serum (LDS) and receiving recombinant ApoE2, ApoE3, ApoE4 or no ApoE, in the presence or in the absence of 4F or scramble 4F (4F-Sc) peptide (See schematic in Figure 5A for the experimental design, and Figure 6A for images of SDS-PAGE followed by ApoE detection used for quantification). Relative quantification of ApoE signal normalized over cell lysate protein concentrations.

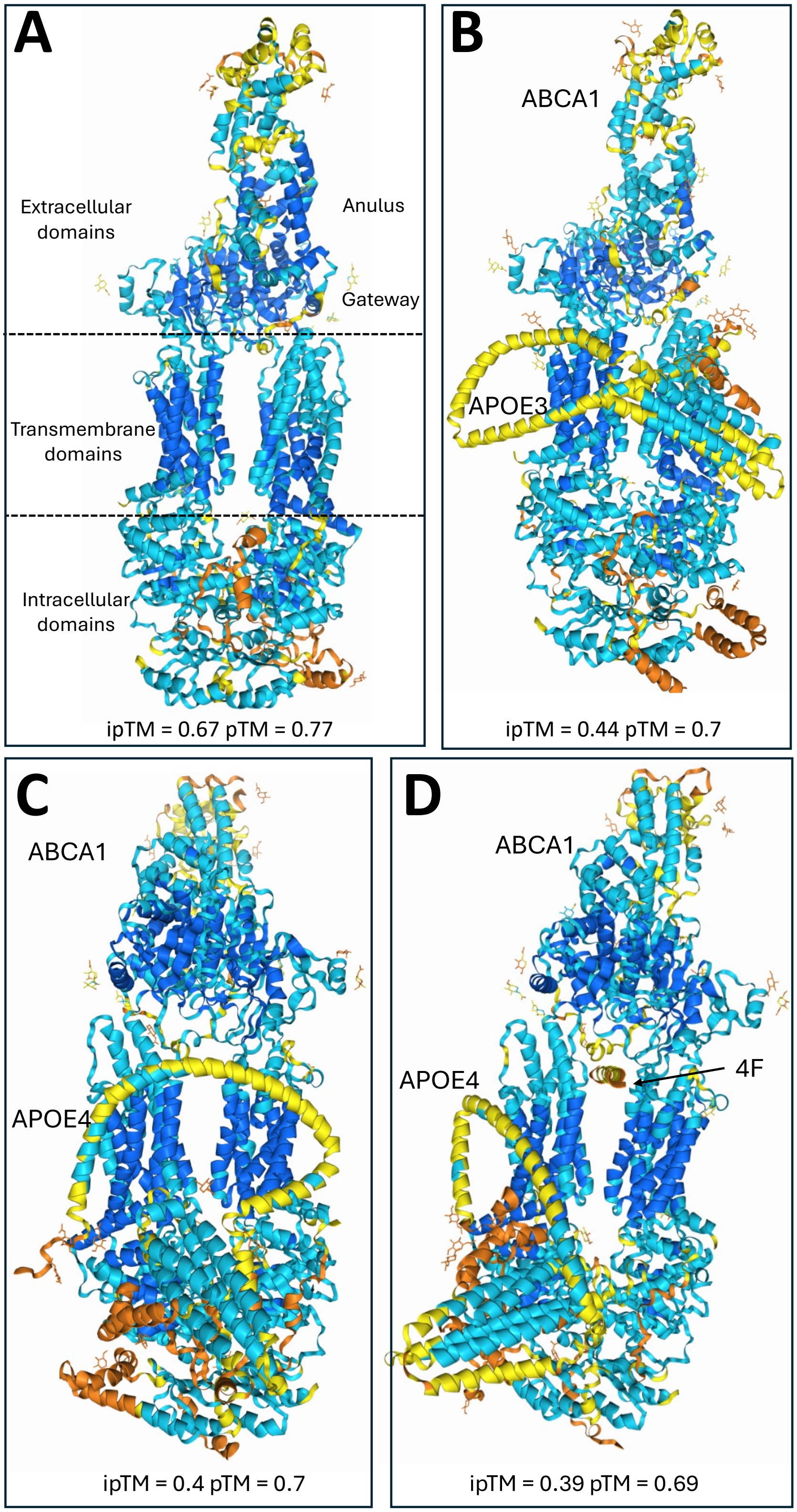

Supplementary Figure 6. In silico modeling of the ABCA1, ApoE4 and 4F protein complex. (A) The predicted protein structure of ABCA1 demonstrated the organization of the extracellular (annulus and gateway domains), transmembrane and intracellular domains. **(B)** Modeling of the ABCA1 and ApoE

protein complex suggested that the N-terminal receptor binding domain of ApoE3 interacted with the transmembrane domains of ABCA1. **(C)** In contrast, the N-terminal receptor binding domain of ApoE4 was predicted to interact with the intracellular domain of ABCA1. **(D)** The 4F lipopeptide was predicted to interact with the transmembrane domains of ABCA1 and altered the interaction of ApoE4 with ABCA1. Abbreviations, ipTM, interface predicted template modeling, pTM, predicted template modeling. Color coded confidence of predicted amino acid conformation; blue = very high confidence, turquoise = confident, yellow = low confidence, orange = very low confidence.

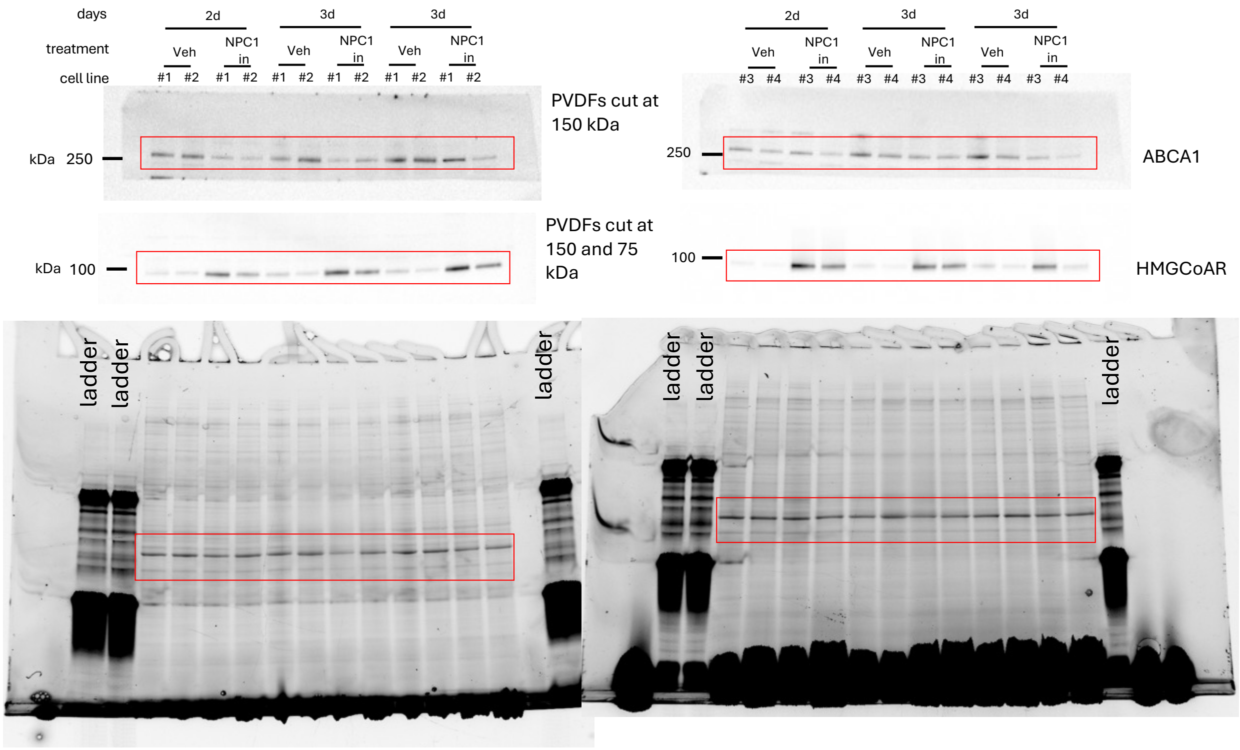

Supplementary Figure 7. Uncropped images of western blot and total protein loading, with red boxes highlighting areas used to create the representative gel image in **Figure 1D**.

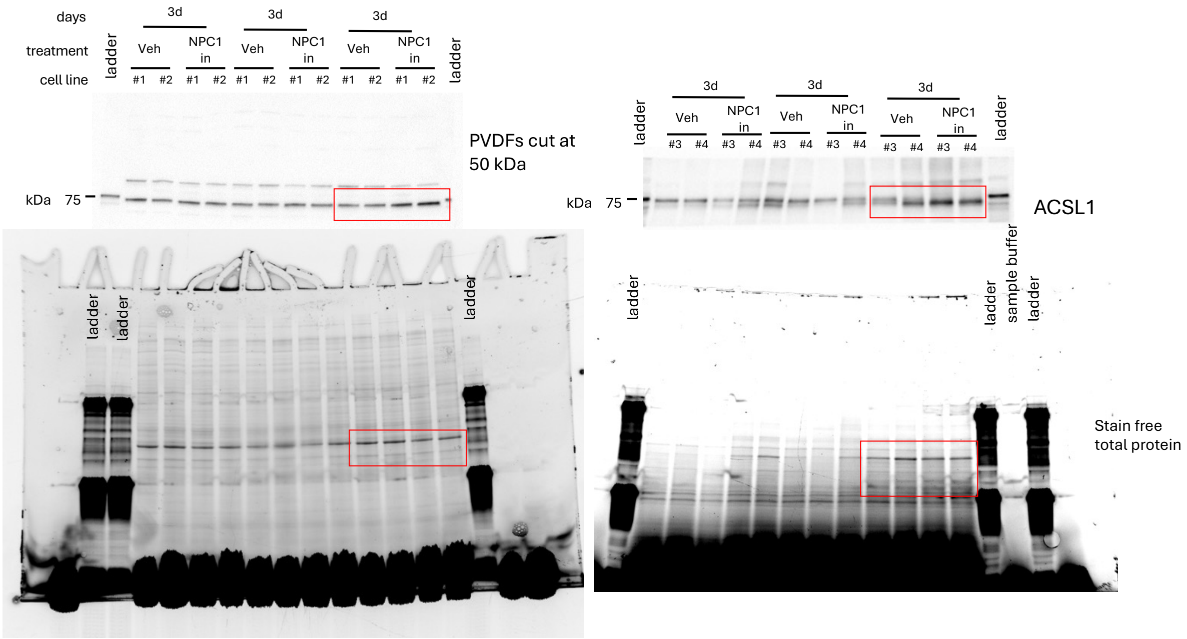

Supplementary Figure 8. Uncropped images of western blots and total protein loading, with red boxes highlighting areas used to create the representative gel image in **Figure 2E**.

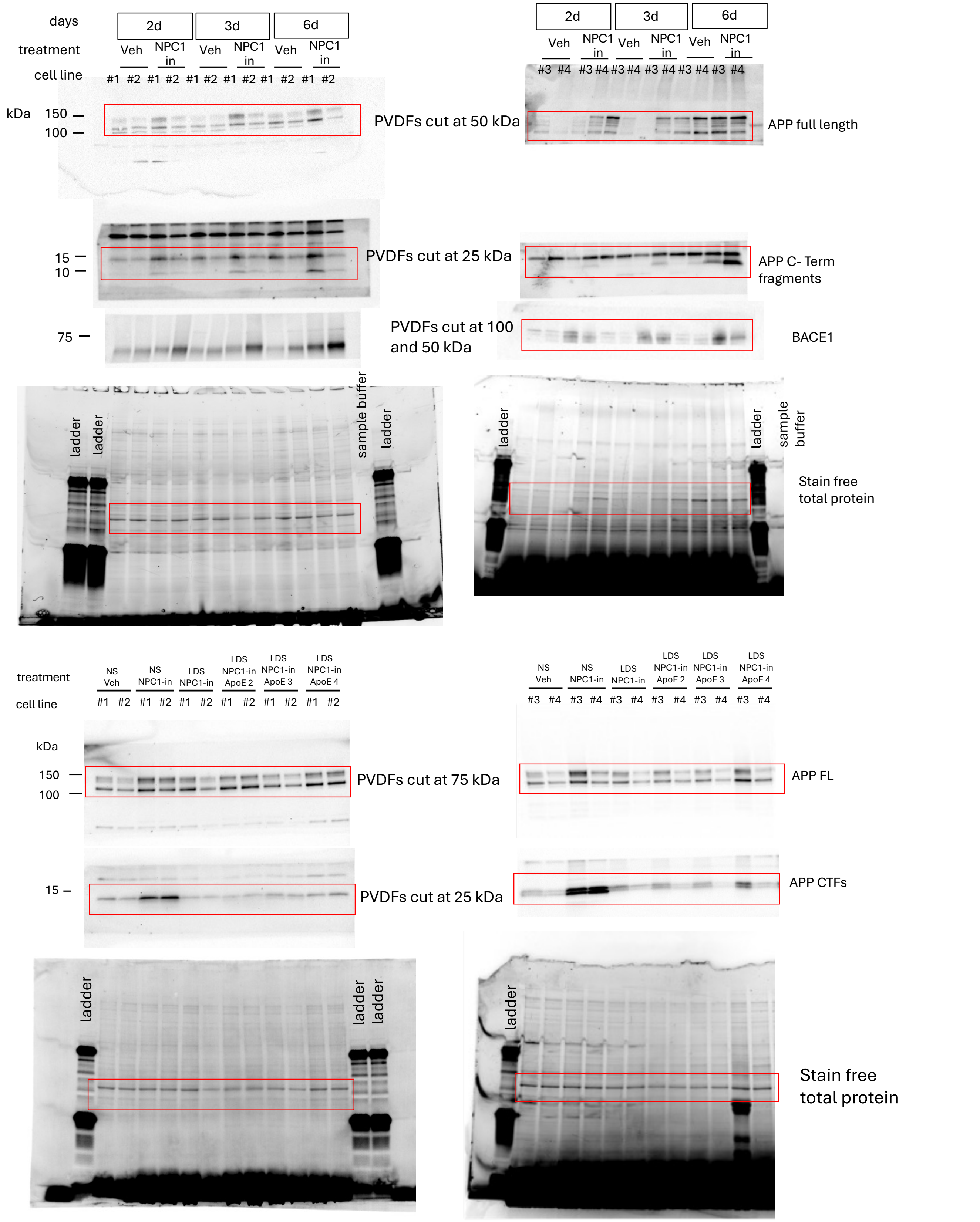

Supplementary Figure 9. Uncropped images of western blots and total protein loading, with red boxes highlighting areas used to create the representative gel images in **Figure 4A** (upper panels) and **4B** (lower panels).

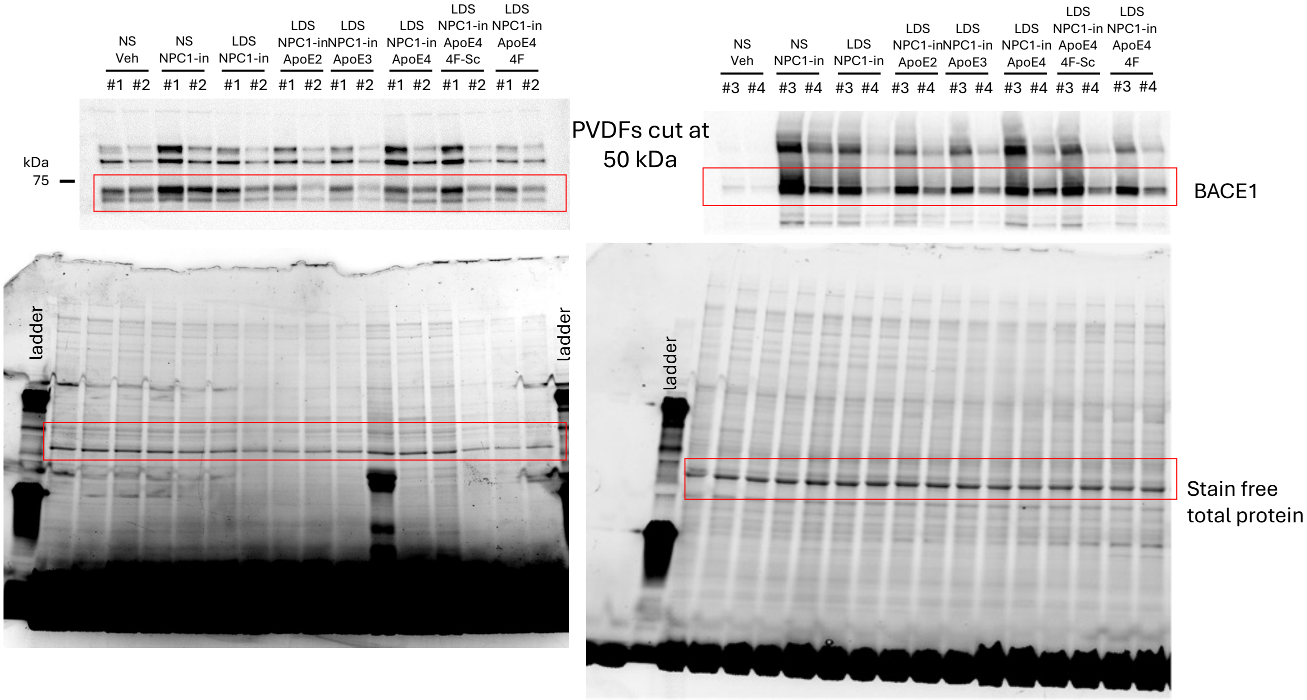

Supplementary Figure 10. Uncropped images of western blots and total protein loading, with red boxes highlighting areas used to create the representative gel image in **Figure 5D**.

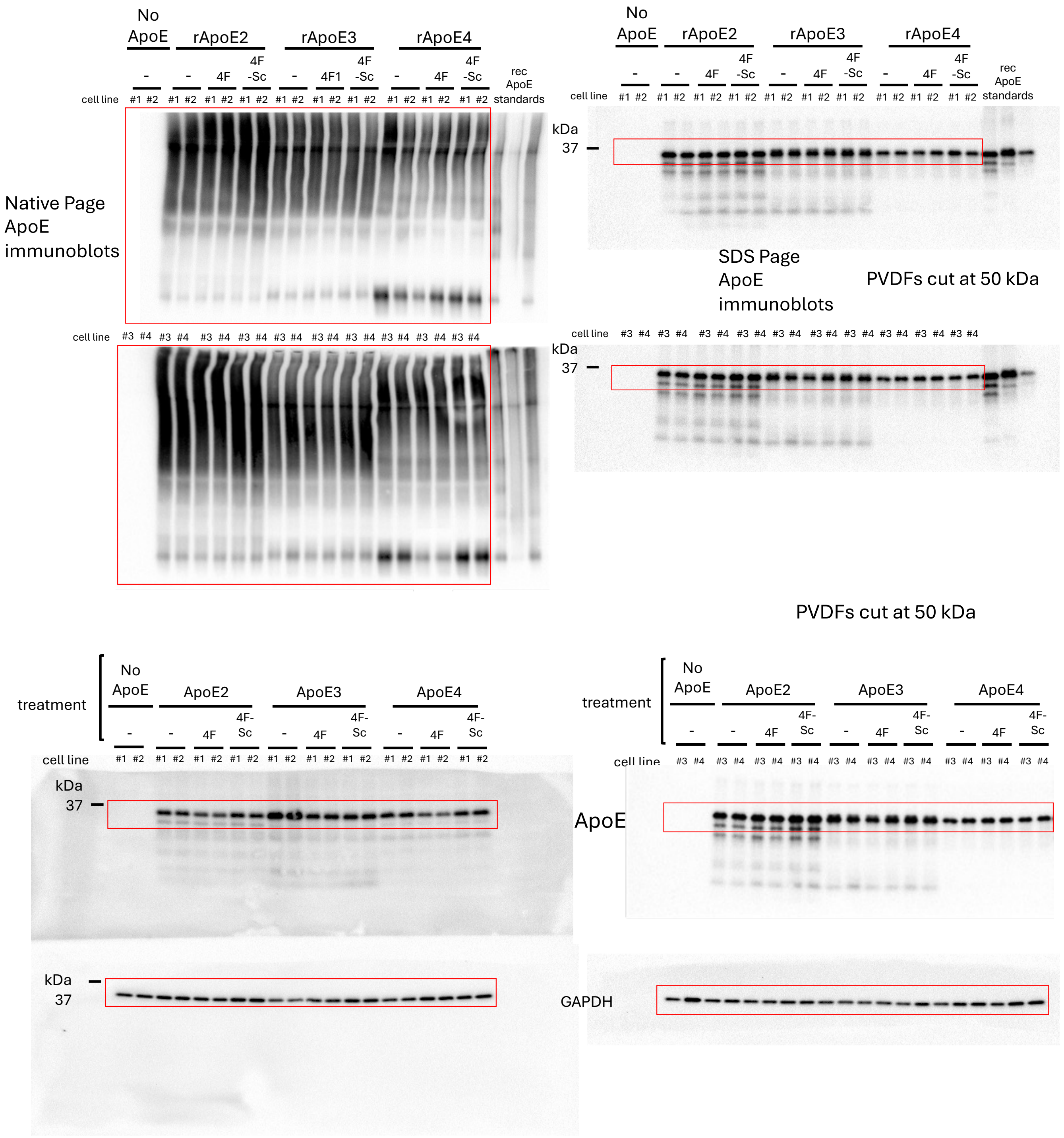

Supplementary Figure 11. Uncropped images of western blots and total protein loading, with red boxes highlighting areas used to create the representative gel images in **Figure 6A** (upper panels) and **6B** (lower panels).

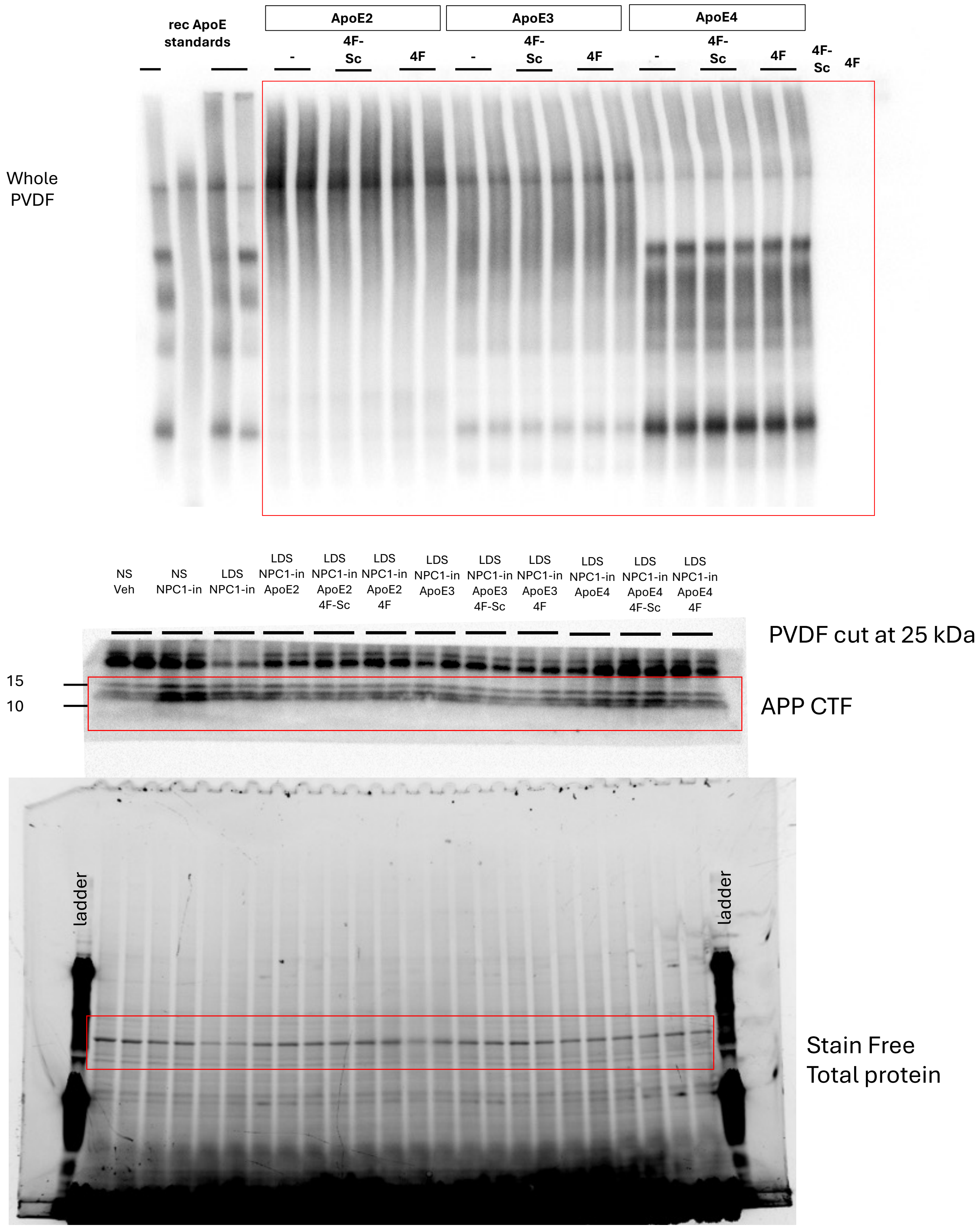

Supplementary Figure 12. Uncropped images of western blots and total protein loading, with red boxes highlighting areas used to create the representative gel images in **Figure 7C** (upper panels) and **7E** (lower panels).
